# Upregulated CD58 is associated with clinicopathological characteristics and poor prognosis of patients with pancreatic ductal adenocarcinoma

**DOI:** 10.1186/s12935-021-02037-0

**Published:** 2021-06-30

**Authors:** Yalu Zhang, Qiaofei Liu, Jingkai Liu, Quan Liao

**Affiliations:** Department of General Surgery, State Key Laboratory of Complex Severe and Rare Diseases, Peking Union Medical College Hospital, Chinese Academy of Medical Science and Peking Union Medical College, Beijing, 100730 China

**Keywords:** CD58, Pancreatic ductal adenocarcinoma, Survival, Prognosis, Immune infiltration

## Abstract

**Background:**

CD58 has been demonstrated to be abnormally expressed in multiple hematopoietic malignancies and solid tumors and plays an essential role in tumorigenesis and progression; however, its clinical significance and prognostic value in pancreatic ductal adenocarcinoma (PDAC) remain unknown.

**Methods:**

Based on diverse online public databases and 81 PDAC samples of tissue microarray-based immunohistochemistry (IHC), we evaluated CD58 expression in PDAC patients and analyzed its association with clinicopathological characteristics, clinical outcomes, and infiltration of immune cells in PDAC. Furthermore, the correlation between CD58 and the cancer stem cell (CSC)-related, epithelial–mesenchymal transition (EMT)-related, and immune-related markers were detected. Besides, the functional enrichment analysis and related pathways were analyzed and visualized.

**Results:**

CD58 expression was elevated in pancreatitis and PDAC tissues than normal pancreas or adjacent nontumor tissues. The positive cases of CD58 (e.g. more than 50% positive cells) in PDAC account for 95.06% (77/81). Upregulated CD58 in cancer tissues was associated with worse histological grade, larger tumor size, and poorer overall survival and disease-free survival in PDAC patients. Furthermore, Cox multivariate regression analysis revealed that CD58 was an independent prognostic factor in PDAC. CD58 expression was correlated with infiltrations of neutrophils, CD8^+^ T cells, and dendritic cells (DCs). In addition, correlation gene analysis indicated that CD58 expression was strongly correlated with immune-related, EMT-related, and CSC-related markers. Functional enrichment analysis and KEGG pathway manifested that CD58 might be involved in PDAC initiation and progression.

**Conclusions:**

CD58 expression is upregulated in PDAC tissues and its high expression is notably related to poor survival of PDAC. Therefore, CD58 may serve as a novel and effective marker for predicting the prognosis of PDAC patients.

**Supplementary Information:**

The online version contains supplementary material available at 10.1186/s12935-021-02037-0.

## Background

Pancreatic ductal adenocarcinoma (PDAC), one of the most aggressive and refractory types of malignancies, accounts for about 90% of all pancreatic cancer cases [1], and is expected to overtake lung cancer as the second leading cause of cancer-related deaths over the next decade [2]. With the highest incidence-to-mortality ratio, the 5-year survival rate of PDAC is only 9% after diagnosis owing to its strong ability in local invasion, early metastasis, as well as drug resistance [3, 4]. Although adjuvant chemotherapy and surgery may provide opportunities to prolong survival, the prognosis of PDAC patients after resection remains unsatisfactory in most cases [5–7]. Therefore, it is of great significance to identify a novel and specific marker to provide an accurate prediction of prognosis for PDAC patients.

CD58 is a member of the immunoglobulin superfamily and is encoded by a gene on chromosome 1 [8]. CD58 is a heavily glycosylated cell adhesion molecule that is broadly distributed on both hematopoietic and nonhematopoietic tissues as a type-I transmembrane or a phosphatidylinositol-anchored form [9, 10]. It serves as a natural ligand for CD2 receptor presented on natural killer (NK) cells and T cells [11, 12]. Cell–cell adhesion is crucial for many immunological functions, such as the interaction of cytotoxic T lymphocytes (CTL) with their targets [13]. T/NK cells can adhere and recognize CD58 molecules of target cells through CD2 molecules on their surface, thus generating costimulatory signaling [8].

In addition to promoting adhesion between cells, the molecular interaction between CD2 and its ligand CD58 has been thought to be involved in lymphocyte activation and effector functions, including cytolytic activity on neoplastic cells [14–16]. Intriguingly, under normal physiological conditions, as non-malignant B cells differentiate from early to mature stages in the bone marrow, CD58 expression was profoundly reduced. However, under pathological conditions, malignant precursor B-cell acute lymphoblastic leukemia cells expressed remarkably higher CD58 levels than non-malignant B cells at any maturational stage. A loss of CD58 might contribute to the escape of neoplastic cells from immune surveillance by CTLs and NK-cell mediated cytolysis [17, 18]. CD58 is markedly decreased, even lost, in many hematological malignancies, including diffuse large B-cell lymphoma, Burkitt’s lymphoma, chronic myelogenous leukemia, acute lymphoid leukemia [19–21]. Its loss was relevant in worse overall survival (OS) and disease-free survival (DFS) in acute lymphoblastic leukemia and diffuse large B-cell lymphoma [22–24]. In contrast, CD58 expression was significantly increased in multiple solid tumors, including gastric cancer, colorectal cancer, and glioblastomas [25–27]. However, CD58 roles in PDAC and its clinical implications in prognosis prediction remain to be investigated.

Herein, we investigated CD58 expression in PDAC tissues, its correlation with clinicopathological characteristics, and prognostic implications of PDAC patients using different public databases and tissue microarray-based immunohistochemistry (IHC). We further explored the association between CD58 expression and infiltrated immune cells in PDAC. Furthermore, the correlation of CD58 with immune-related, EMT-related, CSC-related markers were evaluated. The functional enrichment analysis and related pathways on CD58 were analyzed and visualized.

## Materials and methods

### Patients and specimens

A total of 81 patients who were pathologically diagnosed as PDAC were enrolled. PDAC tissues and paired adjacent nontumor tissues were collected. From January 2008 to June 2011, a follow-up was conducted every 3 to 6 months. Inclusion criteria: (1) older than 18 years; (2) pathologically diagnosed with PDAC; (3) both paired cancer and paracancer tissues were obtained; (4) radical pancreaticoduodenectomy (with or without pylorus preservation). Exclusion criteria: (1) undergo neoadjuvant chemotherapy; (2) pathological specimens could not be obtained; (3) refused follow-up. The detailed clinicopathological records and follow-up information were available for all the patients. The diagnosis and staging were based on the 7th edition of the American Joint Committee on Cancer (AJCC). The male-to-female ratio is 50:31. The age of the patients ranges from 35 to 81 years with a mean age of 59.1 ± 10.3 years. Of the 81 cases, 65 died, and the remaining 16 were still alive until the end of the follow-up period. The median time of follow-up was 13.2 months (range 2.0–41.3). The clinicopathological characteristics of PDAC patients are summarized in Table 1. This study was approved by the Ethics Committee of Peking Union Medical College Hospital. All the patients enrolled in the present study provided written informed consent.

**Table 1:** Relationship between CD58 expression and clinicopathological characteristics in PDAC patients

Parameters	Total	CD58 expression		*p*-value
		Low (n = 41)	High (n = 40)	
Age (years)				
< 60	39	24	15	0.058
≥ 60	42	17	25	
Gender				
Female	31	17	14	0.550
Male	50	24	26	
Histological grade				
G1–2	62	36	26	0.015*
G3	19	5	14	
Tumor location				
Head	53	30	23	0.138
Body/tail	28	11	17	
Tumor size (cm)				
≦ 4	43	27	16	0.020*
> 4	38	14	24	
Lymph node metastasis				
Negative	45	19	26	0.091
Positive	36	22	14	
TNM stage				
I–IIA	39	17	22	0.223
IIB–III	42	24	18	
Perineural invasion				
Negative	29	17	12	0.282
Positive	52	24	28	
Macrovascular invasion				
Negative	56	32	24	0.079
Positive	25	9	16	

*p < 0.05

### Tissue microarray construction and IHC

The pancreatic cancer tissue microarray (ZuoCheng Bio. China) was established to detect the expression level of CD58 in cancer and paracancer tissues by utilizing formalin-fixed and paraffin-embedded blocks. IHC was performed as previously described [28, 29]. The sections were incubated with 1:400 dilutions of rabbit CD58 antibody (AF1689, R&D systems, USA).

### Evaluation of IHC staining

The staining assessment was independently performed by two experienced pathologists who were blinded to clinicopathological and follow-up data. Their concordance rate reached 93.8%. For slices with different scores, a consensus was reached after discussion. IHC score was applied to evaluate the expression level of CD58, and it was calculated by multiplying an intensity score and a proportion score [29, 30]. The intensity score reflected the staining intensity using a scale of 0–3, as follows: 0, negative; 1, weakly positive; 2, moderately positive; 3, strongly positive. The proportion score represented the fraction of positive-stained cells using a scale of 0–4, as follows: 0, none; 1, 1–25%; 2, 26–50%; 3, 51–75%; 4, 76–100%. Specimens with IHC scores higher than the median were defined as high CD58 expression, whereas the others were deemed as low CD58 expression.

### Survival analysis

GEPIA (http://gepia.cancer-pku.cn/) is a user-friendly web tool for analyzing the differential gene expression and patient survival based on GTEx and TCGA databases through a standard processing approach [31]. “Median” was selected as the “Group Cutoff” for survival analysis. The Kaplan–Meier plotter (http://kmplot.com/analysis/) is an online tool for survival analysis to rapidly evaluate the gene expression impact in 21 cancer types [32], including pancreatic cancer. OncoLnc (http://www.oncolnc.org) allows researchers to efficiently investigate survival relevance among 21 cancers. According to the median value, PDAC patients were classified into high-CD58-expression group and low-CD58-expression group, namely 50% vs. 50%.

### Data mining from public databases

The expression levels of CD58 in diverse normal human tissues were acquired from NCBI (BioProject: PRJEB4337) (https://www.ncbi.nlm.nih.gov/gene/) [33]. SurvExpress (http://bioinformatica.mty.itesm.mx/SurvExpress) is a web server for tumor gene expression by using survival analysis [34]. It includes over 130 datasets and 20,000 samples with censored clinical information. The CD58 expression levels of PDAC patients in the datasets were classified into low- and high-risk groups based on the prognostic index. Oncomine (http://www.oncomine.org) owns robust analysis methods and powerful analysis function sets, which can calculate gene expression characteristics and gene set modules from 715 datasets and 86,733 samples for individual researchers [35], THRESHOLD (*p*‑value) < 1E−10, THRESHOLD (fold change) > 2. TIMER (https://cistrome.shinyapps.io/timer) is a user-friendly online tool for comprehensive analysis of immune infiltrates among different tumors [36], including PDAC.

### Identification of differentially expressed genes (DEGs)

LinkedOmics (http://www.linkedomics.org/) is a public database that contains multi-omics data across 32 cancer types [37]. To identify candidate DEGs regarding CD58, RNAseq data including billions of attribute pairs from 178 PDAC patients were analyzed. Furthermore, the top 10 negatively and positively correlated significant genes were screened, respectively, and the top 500 positive genes were used to perform functional enrichment analysis.

### Functional enrichment analysis

DAVID (https://david.ncifcrf.gov/) was utilized to administrate KEGG pathway and gene ontology (GO) analysis, including biological process (BP), cellular component (CC), and molecular function (MF) [38–40] for functional enrichment analysis of the top 500 DEGs. GeneMANIA (http://genemania.org/) [41], a flexible and valuable platform for prioritization and prediction of gene function, was applied to predict the function of CD58 and its associated networks with other genes.

### Statistical analysis

Graphs and statistical analysis were administrated using GraphPad Prism 6.0 (Lajolla, CA, USA) and IBM SPSS Statistics 21.0 (SPSS Inc., Chicago, USA), respectively. Paired Wilcoxon test was utilized to compare CD58 staining between paracancer normal tissues and tumor tissues. The Pearson Chi-square test was applied to evaluate the correlation between CD58 and clinical parameters. The log-rank test was employed to detect the survival analysis. The Cox proportional hazards regression model was utilized to analyze the multivariable analysis of prognostic factors. A two-tailed *p*-value < 0.05 was considered statistically significant.

## Results

### CD58 was upregulated in pancreatitis and pancreatic cancer tissues by bioinformatics analysis

**Fig. 1:**
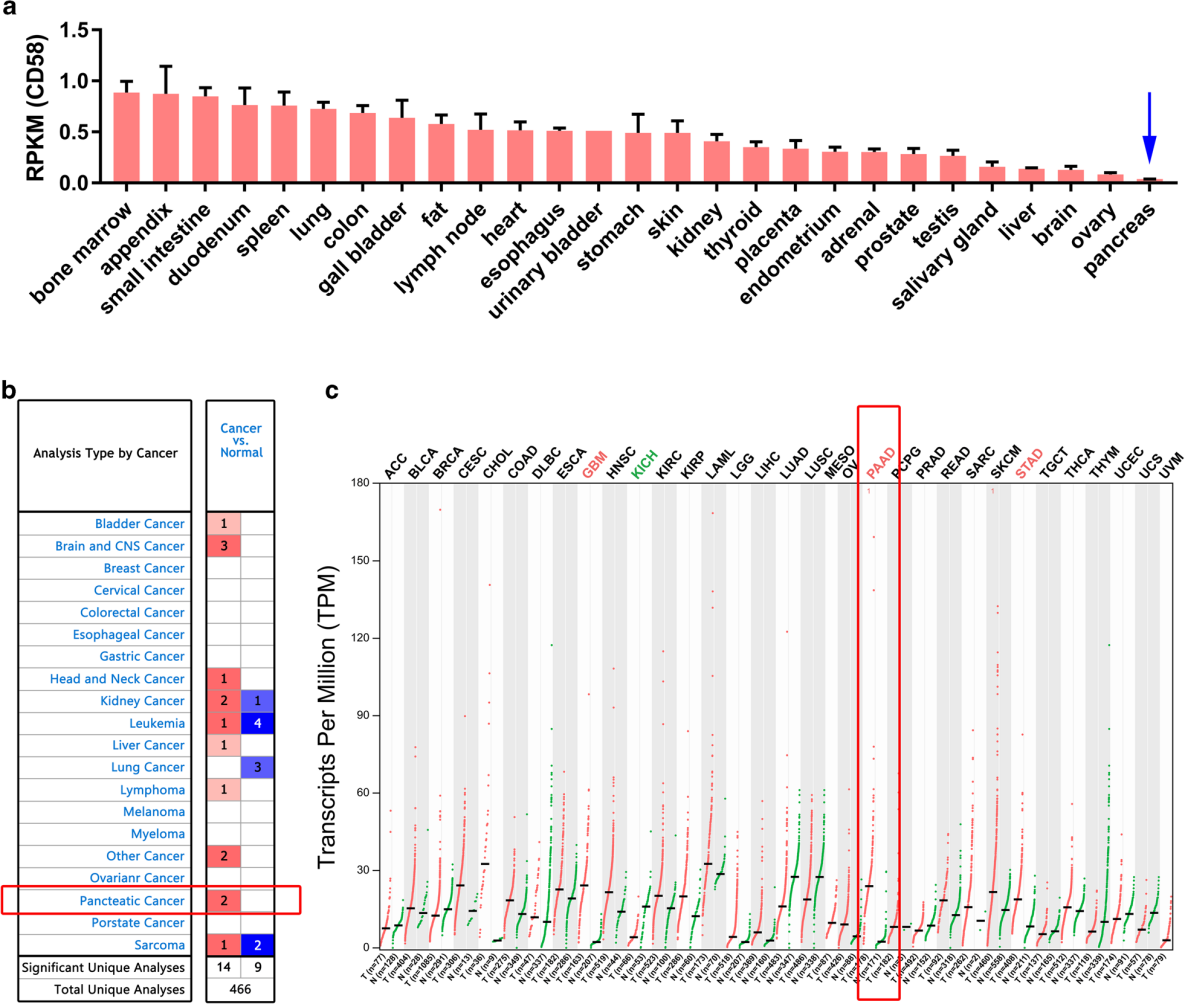
CD58 expression in normal pancreatic tissue and various human cancers. **a** CD58 expression was extremely low in normal pancreatic tissue among the 27 human tissues (data from NCBI, BioProject: PRJEB4337). RPKM, reads per kilobase per million. **b**, **c** Oncomine and GEPIA online tool showed that CD58 was remarkably aberrant expressed in various human cancers, including pancreatic cancer. In Oncomine, red squares represent a high expression of CD58 in tumor tissue, while blue squares represent its low expression. The digit represents the number of relevant datasets. In GEPIA, the red font represents a high expression of CD58 in tumor tissue, while the green font represents its low expression. *KICH* kidney chromophobe cancer, *GBM* glioblastoma multiforme, *STAD* stomach adenocarcinoma, *PAAD* pancreatic adenocarcinoma

The expression level of CD58 gene varies in different normal tissues or organs. The data from NCBI indicated that CD58 expression was extremely low in normal pancreatic tissues than the 27 human tissues or organs (Fig. 1a), including heart, kidney, liver, lung, stomach, spleen, small intestine, thyroid, lymph node, and so on [33]. Analysis from Oncomine, a powerful data analysis platform with 715 datasets and 86,733 samples, found that CD58 was abnormally expressed in multiple human cancers. Among them, more datasets support the lower expression of CD58 in leukemia, lung cancer, and sarcoma, while its expression was significantly increased in bladder cancer, brain and CNS cancer, and pancreatic cancer, compared with corresponding normal tissues (Fig. 1b). By comparing the tumor tissues and corresponding normal samples using GEPIA, the results demonstrated that CD58 level was reduced in kidney chromophobe cancer (KICH). Simultaneously, its expression was observably elevated in glioblastoma multiforme (GBM), stomach adenocarcinoma (STAD), and pancreatic adenocarcinoma (PAAD) (Fig. 1c). Therefore, CD58 might be an effective tumor marker for PDAC.

**Fig. 2:**
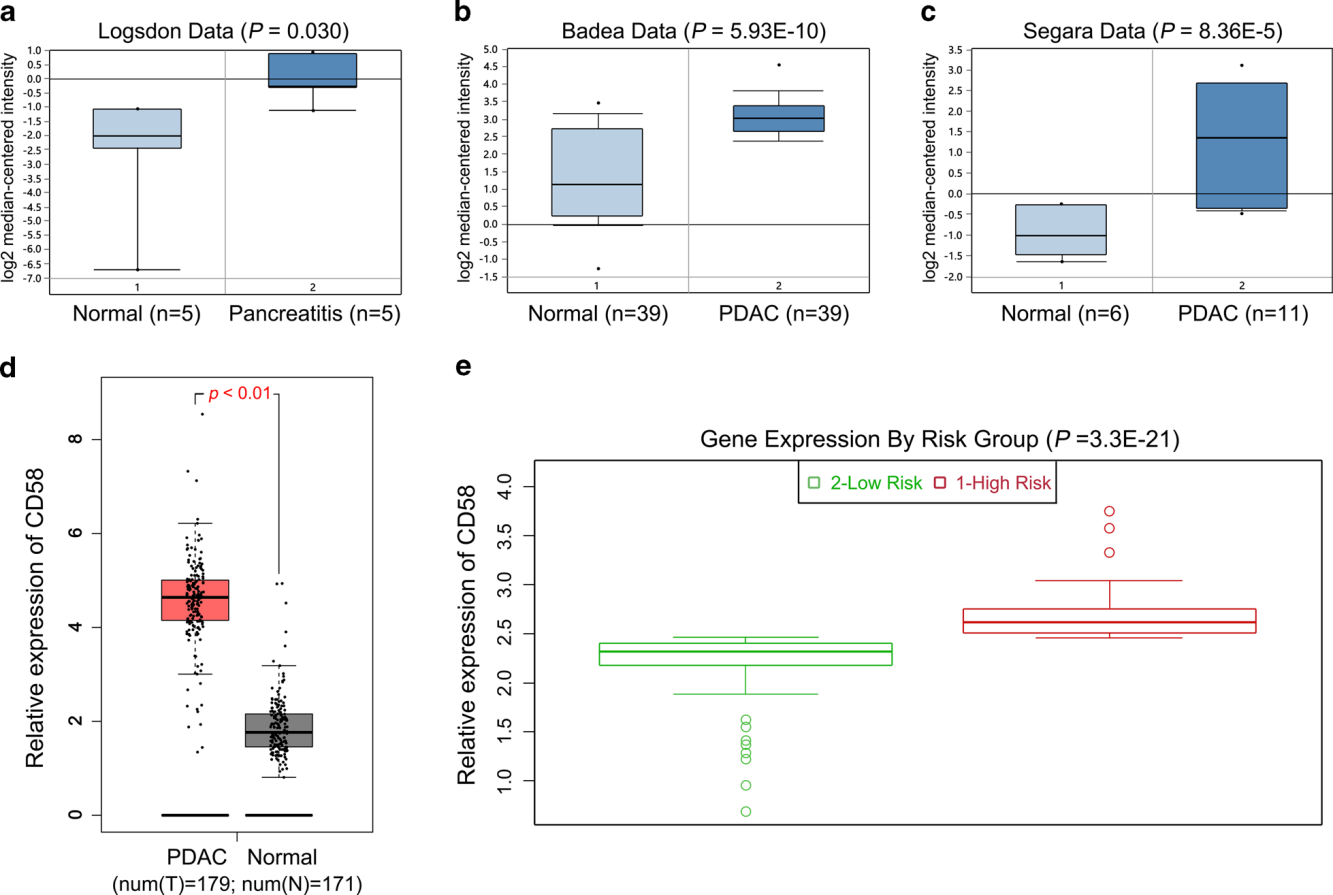
CD58 level in pancreatitis and pancreatic cancer tissues by bioinformatics analysis. **a** Logsdon’ data showed that CD58 was upregulated in pancreatitis tissues (n = 5) compared with the normal pancreas tissues (n = 5), *p* = 0.030. **b**, **c** Bedea and Segara’s data displayed that CD58 was increased in PDAC tissues relative to the normal pancreas tissues. **d** GEPIA online tool confirmed this finding by analyzing TCGA and GTEx databases. N (normal) = 171; N (tumor) = 179; *p* < 0.01. *PAAD* pancreatic adenocarcinoma. **e** After dividing 176 pancreatic cancer patients into a high-risk group and a low-risk group according to the prognosis index, SurvExpress platform showed that the level of CD58 in the high-risk group (n = 88) was higher than that in the low-risk group (n = 88), *p* < 0.001

We also further explored CD58 expression in pancreatic cancer in Oncomine, GEPIA, and SurvExpress. In Oncomine, Logsdon’s data revealed that CD58 expression was notably enhanced in pancreatitis than the normal pancreas tissues (*p* = 0.030) (Fig. 2a); Badea and Segara’s data manifested that CD58 level was markedly elevated in PDAC tissues than normal pancreas tissues (*p* < 0.001 for both) (Fig. 2b, c). In GEPIA, TCGA and GTEx data also indicated that CD58 expression was potently enhanced in PDAC tissues (N = 179) relative to normal pancreas samples (N = 171) (*p* < 0.010, Fig. 2d). According to the prognostic index in SurvExpress, 176 PDAC patients were classified into two groups, namely low- or high-risk groups. The analysis revealed that high-risk group had a higher CD58 expression (*p* < 0.001, Fig. 2e).

### CD58 was markedly associated with poor prognosis through analyzing different databases

**Fig. 3:**
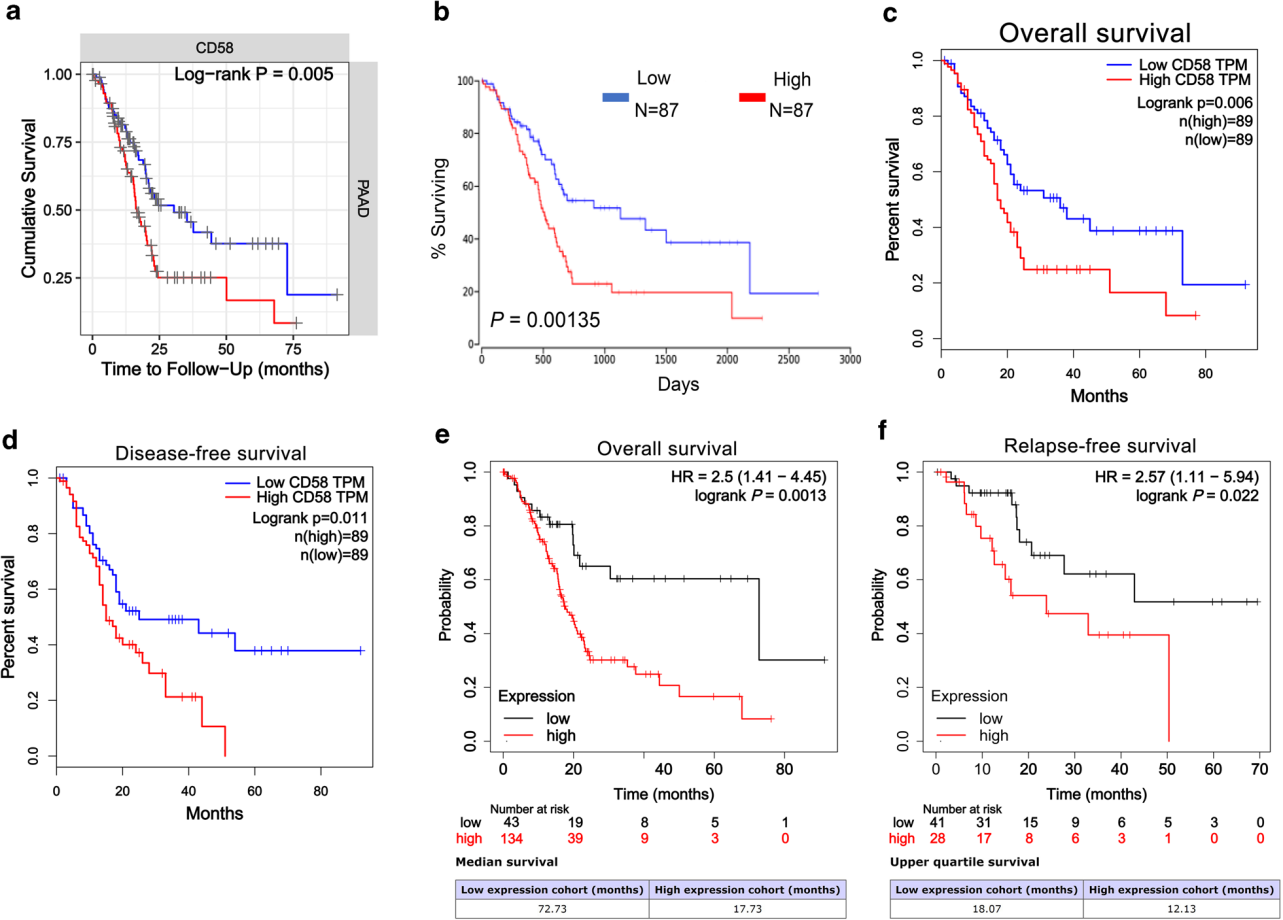
The effect of CD58 in the prognosis of PDAC patients through analyzing different databases. **a**, **b** TIMER and OncoLnc platform demonstrated that high CD58 expression was an adverse prognostic factor for PDAC patients (*p* = 0.005 and 0.00135, respectively). **c**, **d** GEPIA showed that CD58 was not only significantly related to the OS of patients (*p* = 0.006), but also a negative factor for DFS in PDAC (*p* = 0.011). *TPM* transcripts per million. **e**, **f** Kaplan–Meier plotter online tool showed a similar result (*p* = 0.0013 and 0.022, respectively)

Subsequently, we investigated the effect of CD58 expression on pancreatic cancer prognosis using bioinformatics databases, including TIMER, OncoLnc, GEPIA, and Kaplan–Meier plotter. The TIMER and OncoLnc databases manifested that high CD58 expression was associated with poor OS in PDAC patients (*p* = 0.005 and 0.00135, respectively) (Fig. 3a, b). Besides, GEPIA data suggested that CD58 level not only related to OS (*p* = 0.006) but also strongly correlated with DFS (*p* = 0.011, Fig. 3c, d). Similar results could be found in Kaplan–Meier plotter, where PDAC patients with high CD58 expression predicted a worse prognosis for OS (*p* = 0.0013) and relapse-free survival (RFS) (*p* = 0.0220, Fig. 3e, f). Patients with high CD58 expression had 2.5 times the risk of OS than those with low CD58 expression, and the median OS time was 72.73 months for low expression cohort and 17.73 months for high expression cohort (Fig. 3e). Patients with high CD58 level had 2.57 times the risk of RFS versus those with low expression, and the upper quartile survival time of low expression cohort and high expression cohort was 18.07 months and 12.13 months, respectively (Fig. 3f).

### Correlations between CD58 expression and clinicopathological characteristics

**Fig. 4:**
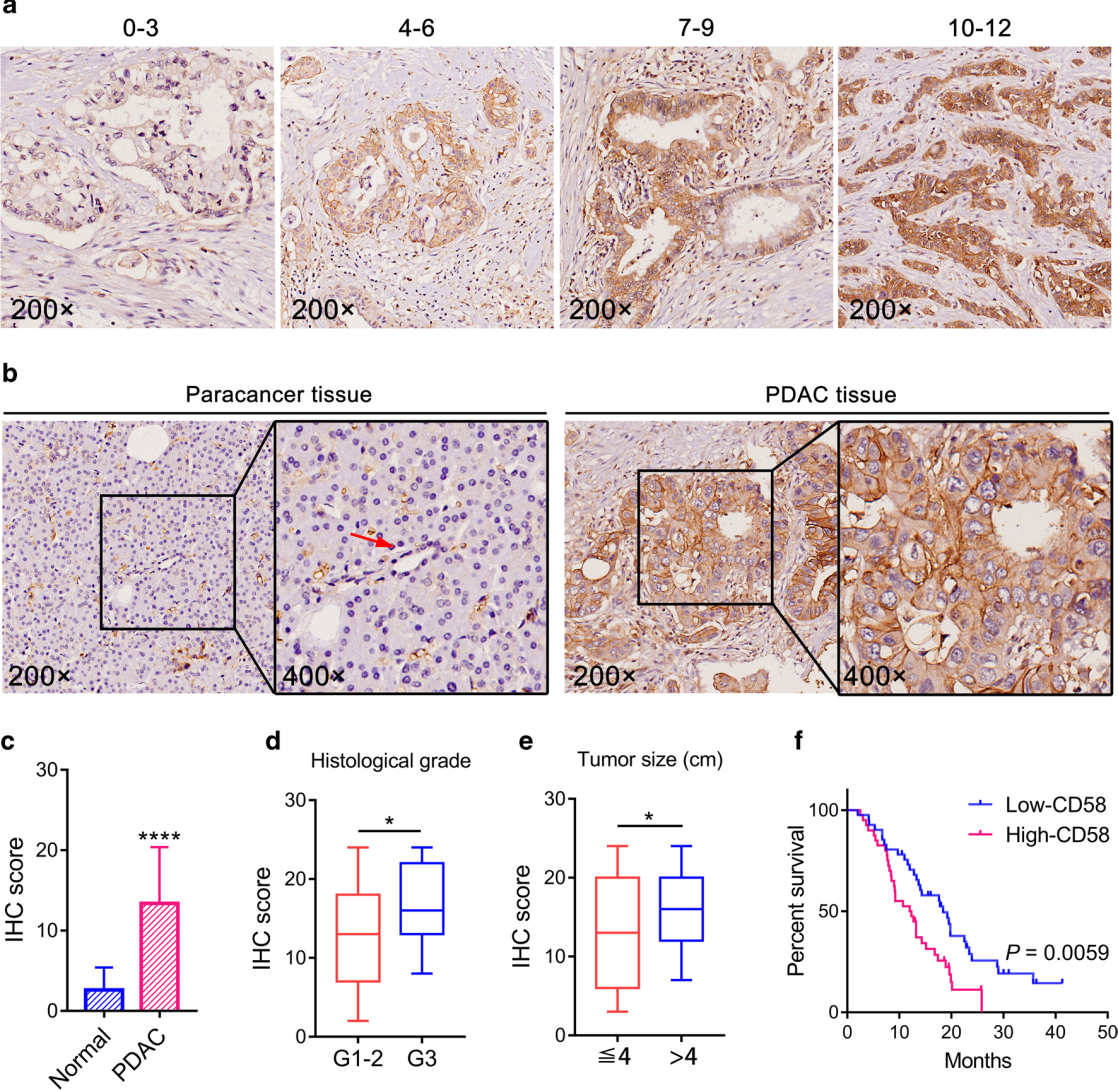
CD58 expression in PDAC tissues and para-cancer normal tissues by using tissue microarray-based IHC. **a** Different IHC scores of CD58 in the PDAC tumor tissues. The cellular staining was classified using a scale of 0–12 (original magnification, 200×). **b** Representative microphotographs of normal PDAC tissues and paracancer normal tissues. **c** The IHC score of PDAC was higher than that of paracancer normal tissues (Paired Wilcoxon test; *p* < 0.0001). **d**, **e** The expression of CD58 was higher in PDAC patients with higher histological grade (*p* < 0.05) or larger tumor size (*p* < 0.05). **f** Log-rank test revealed that the high-CD58-group had a worse prognosis than low-CD58-group in patients with PDAC (*p* = 0.0059)

To further verify the above findings, we used PDAC tissue microarray to conduct IHC staining for CD58 on tumor tissues and paired adjacent normal tissues. The different IHC scores were evaluated according to diverse staining intensities and extents of CD58 (Fig. 4a). As depicted in Fig. 4b, the level of CD58 staining was remarkably higher in PDAC tissues than adjacent normal tissues. The median IHC score of CD58 was 5.5 (range, 0–12). The IHC score of CD58 was notably higher in PDAC tissues than in paracancer tissues (*p* < 0.0001, Fig. 4c). Furthermore, CD58 expression was positively associated with histological grade (*p* = 0.015) and tumor size (*p* = 0.020), namely the higher the pathological grade and the larger the tumor, the higher the expression of CD58 (Table 1 and Fig. 4d, e), but no significant correlation was detected between CD58 expression and other clinicopathological parameters, including age, gender, tumor location, lymph node metastasis, TNM stage, perineural invasion, and macrovascular invasion.

Kaplan–Meier method and log-rank test were utilized to detect the effect of CD58 expression on OS of PDAC patients. The analysis confirmed that patients with high CD58 expression had the worse prognosis (*p* = 0.0059, Fig. 4f). The median survival time of low-CD58- and high-CD58-groups was 18.4 months and 12.0 months, respectively.

### CD58 was an independent prognostic factor in PDAC patients

The univariate analysis indicated that poor OS of patients was relevant in high CD58 expression (*p* = 0.006), age (*p* = 0.007), gender (*p* = 0.026), tumor size (*p* = 0.045), TNM stage (*p* = 0.040) and perineural invasion (*p* = 0.013) (Table 2). Next, we incorporated indicators with *p*-value less than 0.05 into Cox multivariate analysis model. The analysis demonstrated that age and CD58 were independent prognostic factors (*p* = 0.014 and 0.045, respectively) (Table 2).

**Table 2:** Univariate and multivariate analyses for prognostic factors of PDAC patients

Parameters	Univariate analysis		Multivariate analysis	
	HR (95% CI)	*p*-value	HR (95% CI)	*p*-value
Age (years)				
< 60 vs ≥ 60	1.984 (1.198–3.285)	0.008*	1.949 (1.144–3.320)	0.014*
Gender				
Female vs male	1.826 (1.066–3.128)	0.028*	1.571 (0.901–2.737)	0.111
Histological grade				
G1–2 vs G3	1.11 (0.629–1.958)	0.718		
Tumor location				
Head vs body/tail	0.935 (0.558–1.567)	0.798		
Tumor size (cm)				
≦ 4 vs > 4	1.649 (1.004–2.709)	0.048*	1.284 (0.747–2.210)	0.366
Lymph node metastasis				
Negative vs positive	1.508 (0.923–2.463)	0.101		
TNM stage				
I–IIA vs IIB–III	1.664 (1.018–2.721)	0.042*	1.576 (0.913–2.719)	0.102
Perineural invasion				
Negative vs positive	1.944 (1.139–3.320)	0.015*	1.636 (0.909–2.945)	0.101
Macrovascular invasion				
Negative vs positive	1.557 (0.922–2.628)	0.098		
CD58 expression				
Low vs high	2.027 (1.212–3.389)	0.007*	1.796 (1.013–3.185)	0.045*

*HR* hazard ratio, *CI* confidence interval

*p < 0.05

**Fig. 5:**
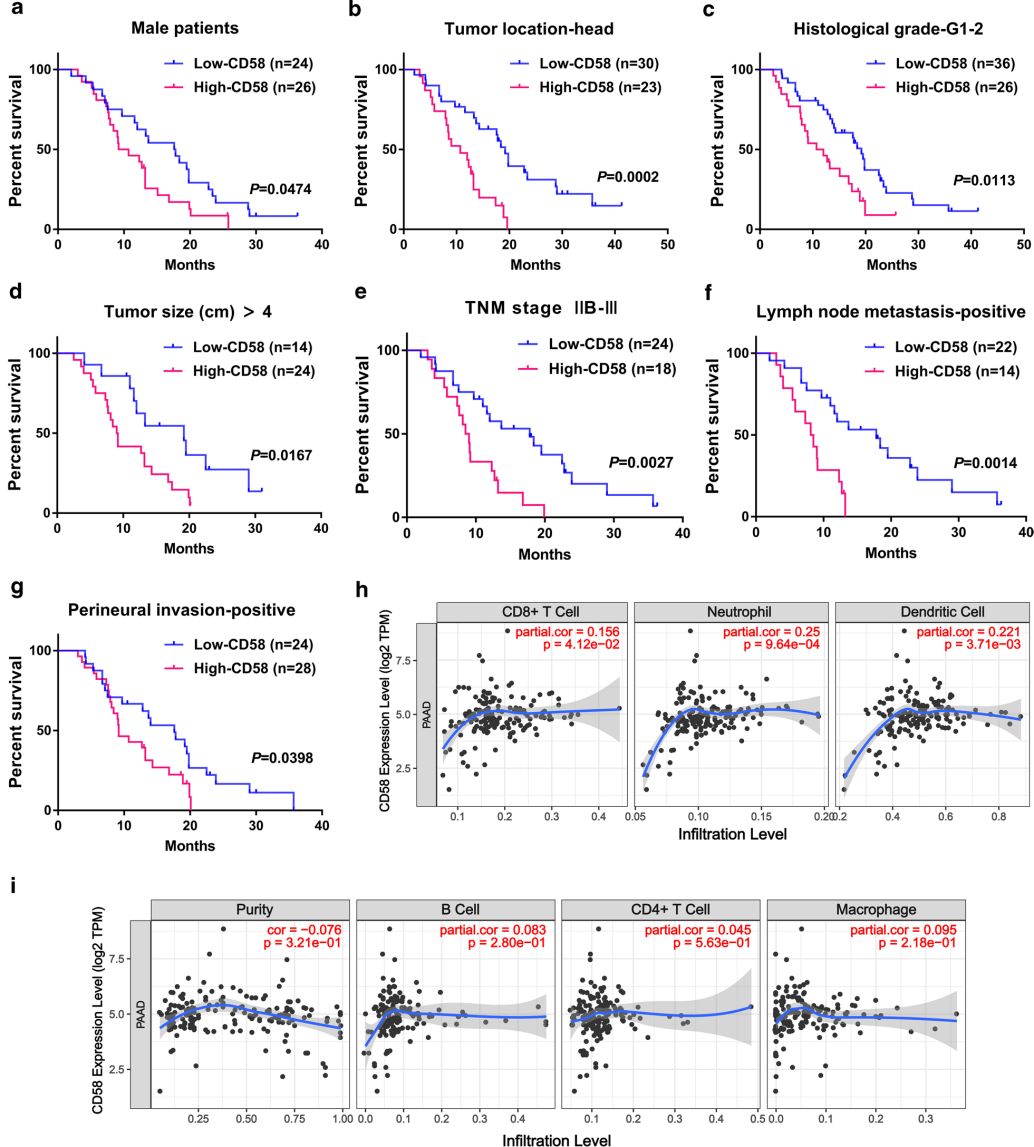
Kaplan–Meier survival analysis of the PDAC patients based on the CD58 expression in many subgroups. **a** Male patients (*p* = 0.0474); **b** The head of the pancreas (*p* = 0.0002); **c** G1–2 tumors (*p* = 0.0113); **d** Tumor size > 4 cm (*p* = 0.0167); **e** TNM stage IIB–III (*p* = 0.0027); **f** Lymph node metastasis-positive (*p* = 0.0014); **g** Nerve infiltration-positive (*p* = 0.0398). G1, well differentiated; G2, moderately differentiated. **h**, **i** Correlation between CD58 and infiltrated immune cell in PDAC by using TIMER. TPM, transcripts per million

Additionally, the subgroup analysis of PDAC patients suggested that CD58 expression in cancer tissues was a powerful prognostic marker in male (*p* = 0.0474), pancreatic head carcinoma (*p* = 0.0002), histological G1–2 (*p* = 0.0113), tumor size > 4 cm (*p* = 0.0167), TNM stage IIB–III (*p* = 0.0027), lymph node metastasis-positive (*p* = 0.0014) and perineural invasion-positive group (*p* = 0.0398) (Fig. 5a–g, Table 3).

**Table 3:** The prognostic relevance of CD58 expression in PDAC subgroups in which the poorer overall survival of patients is significantly associated with high CD58 expression

Subgroups	HR	95% CI	*p*-value
Male	1.740	0.96–3.15	0.0474
Head of pancreas	2.730	1.38–5.40	0.0002
G1–2	1.993	1.06–3.74	0.0113
Tumor size > 4 cm	2.323	1.16–4.65	0.0167
TNM stage IIB–III	2.485	1.19–5.21	0.0027
Lymph node metastasis-positive	2.869	1.19–6.90	0.0014
Perineural invasion-positive	1.762	0.97–3.21	0.0398

*HR* hazard ratio, *CI* confidence interval

### Correlation of CD58 with immune cell infiltration, CSC-related genes, and EMT-related genes

As an immune-related molecule, CD58 may play a role in tumor immune microenvironment. Accordingly, we further explored the relationship between CD58 and infiltrations of immune cells in PDAC. TIMER is a friendly online tool with a gene module that enables investigators to choose any gene of interest and visualize the correlation between gene expression and immune infiltration of multiple tumor types. TIMER database revealed that CD58 expression was strongly related to the infiltration of CD8^+^ T cells (*p* = 0.0412), neutrophils (*p* < 0.001) and DCs (*p* < 0.01) (Fig. 5h), but not related to infiltrations of B cells, CD4^+^ T cells and macrophages (Fig. 5i) in PDAC. Furthermore, we also analyzed the correlation of CD58 with immune cell-associated markers using RNA-seq data from 178 PDAC patients in GEPIA and TIMER databases (Table 4, Additional file 1: Figure S1, Additional file 2: Figure S2). The results showed that CD58 expression was positively linked to the monocyte marker CD86, neutrophil markers CD66b and CD11b, tumor-associated macrophage (TAM) marker CD68. In contrast, CD58 expression was negatively linked to NK cell marker CD56 and DC marker S100.

**Table 4:** The correlation of CD58 with immune-related, EMT-related, CSC-related markers

Types	Markers	TIMER		GEPIA	
		R	*p*-value	R	*p*-value
Immune cell	CD45 (PTPRC)	0.050	0.510	0.037	0.620
T cell (general)	CD3D	− 0.008	0.911	− 0.026	0.730
	CD3E	− 0.002	0.982	− 0.013	0.860
	CD3G	0.027	0.721	− 0.028	0.710
CD4^+^ T cell	CD4	0.088	0.242	0.075	0.320
	FOXP3	0.057	0.451	0.066	0.380
CD8^+^ T cell	CD8A	− 0.001	0.986	− 0.200	0.790
	CD8B	0.019	0.799	0.009	0.910
B cell	CD19	0.030	0.695	0.022	0.770
	CD79A	− 0.018	0.814	− 0.046	0.540
Monocyte	CD86	0.158	*	0.150	*
	CD115 (CSF1R)	0.043	0.568	0.053	0.480
Neutrophil	CD66b (CEACAM8)	0.182	*	0.190	*
	CD11b (ITGAM)	0.255	***	0.240	**
	CCR7	0.013	0.860	− 0.003	0.97
TAM	CD68	0.288	***	0.290	***
	CCL2	− 0.053	0.477	− 0.051	0.500
M1 Macrophage	iNOS (NOS2)	0.086	0.249	0.110	0.130
	HLA-DRB1	0.067	0.375	0.089	0.230
M2 Macrophage	CD163	0.088	0.243	0.110	0.140
	CD204 (MSR1)	0.138	0.067	0.140	0.057
	CD206 (MRC1)	0.066	0.382	0.058	0.440
NK cell	CD56 (NCAM1)	− 0.353	***	− 0.230	**
	KIR2DL1	− 0.043	0.564	− 0.050	0.510
	KIR3DL1	− 0.106	0.158	− 0.130	0.081
	KIR2DS4	0.036	0.633	0.027	0.720
DC	S100A1	− 0.148	*	− 0.160	*
	S100B	− 0.030	0.686	− 0.028	0.710
EMT-related	Vimentin (VIM)	0.196	**	0.210	**
	E-Cadherin (CDH1)	0.506	***	0.540	***
	Twist (TWIST1)	0.313	***	0.320	***
	Snail (SNAI1)	0.157	*	0.170	*
	N-Cadherin (CDH2)	0.032	0.670	0.051	0.500
CSC-related	CD133 (PROM1)	0.334	***	0.350	***
	OCT4 (POU5F1)	0.407	***	0.420	***
	KLF4	0.377	***	0.400	***
	NANOG	0.060	0.428	0.170	*
	CD24	0.149	*	0.140	0.059
	ALDH1A1	− 0.044	0.560	− 0.047	0.530
	SOX2	− 0.032	0.673	− 0.021	0.780

N = 178

*R* Spearman’s rho value, *TAM* tumor-associated macrophage, *NK cell* natural killer cell, *EMT* epithelial–mesenchymal transition, *CSC* cancer stem cell

*p < 0.05, **p < 0.01, ***p < 0.001

As an adhesion molecule, CD58 may mediate cell–cell or cell–matrix adhesion and play a role during EMT. Consequently, we explored the correlation of CD58 with EMT-related genes (Table 4, Additional file 1: Figure S1, Additional file 2: Figure S2). The results indicated that CD58 expression was significantly relevant in the expression of Vimentin, E-Cadherin, Twist, and Snail. Moreover, it has been reported that CD58 expression was linked to cell stemness [26]; therefore, we investigated the correlation of CD58 with stem cell markers in PDAC (Table 4, Additional file 1: Figure S1, Additional file 2: Figure S2). The results illustrated that CD58 expression was strongly correlated with CD133, OCT4, and KLF4. Overall, these findings implied that CD58 is critical in immune cell infiltration, EMT, and CSC in PDAC.

### DEGs of CD58 and associated pathways in PDAC

**Fig. 6:**
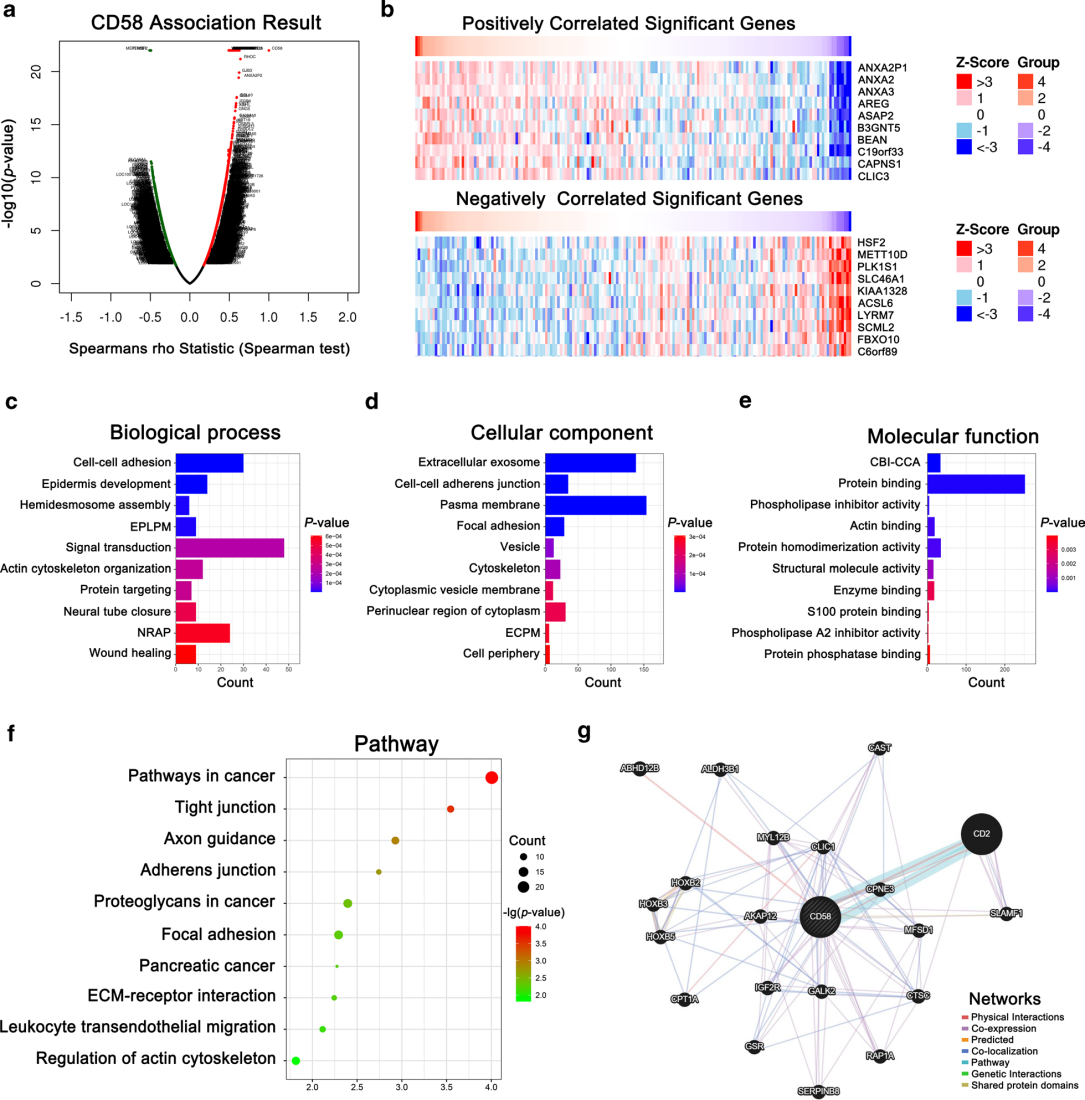
DEGs of CD58 and associated pathways in PDAC. **a** The DEGs of CD58 were visualized by a volcano map. **b** The heatmaps showed the top 10 positively and negatively correlated significant genes, respectively. **c** Bio biological process (BP), **d** Cellular component (CC), **e** molecular function (MF) of gene ontology (GO).** f** The related signaling pathway of CD58 was performed by KEGG pathway analysis. *EPLPM* establishment of protein localization to plasma membrane, *NRAP* negative regulation of apoptotic process, *ECPM* extrinsic component of plasma membrane, *CBI-CCA* cadherin binding involved in cell–cell adhesion. **g** The function of CD58 and related networks were predicted using geneMANIA

To investigate the biological roles of CD58 in PDAC, the DEGs of CD58 were analyzed and visualized. The volcano map showed the identified DEGs (Additional file 3: Table S1) and the heatmaps exhibited the top 10 positively and negatively associated significant genes, respectively (Fig. 6a, b). The top 10 positively associated genes identified were ANXA2P1, ANXA2, ANXA3, AREG, ASAP2, B3GNT5, BEAN, C19orf33, CAPNS1, and CLIC3. The top 10 negatively associated genes were HSF2, METT10D, PLK1S1, SLC46A1, KIAA1328, ACSL6, LYRM7, SCML2, FBXO10, and C6orf89.

To acquire a better understanding of the function and relationship concerning CD58-related genes, we selected the top 500 positively correlated genes and conducted the functional enrichment analysis using DAVID (Additional file 4: Table S2). Regarding the GO analysis, BP terms were implicated in cell–cell adhesion, epidermis development, hemidesmosome assembly, establishment of protein localization to plasma membrane, and signal transduction (Fig. 6c). CC terms were involved in extracellular exosome, cell–cell adherens junction, plasma membrane, focal adhesion, vesicle, and cytoskeleton (Fig. 6d). The MF terms indicated that cadherin binding involved in cell–cell adhesion, protein binding, phospholipase inhibitor activity, actin-binding, and protein homodimerization activity (Fig. 6e). Furthermore, the KEGG pathway analysis to explore CD58-related signaling pathways indicated the pathways in cancer, tight junction, axon guidance, adherens junction, proteoglycans in cancer, focal adhesion, pancreatic cancer, ECM-receptor interaction, and leukocyte transendothelial migration, as well as regulation of actin cytoskeleton (Fig. 6f). The function of CD58 and related networks were predicted using geneMANIA (Fig. 6g). We found that, in addition to interacting with CD2, CD58 was co-expressed and physically interacted with HOXB family genes (HOXB2, HOXB3, and HOXB5), AKAP12, CLIC1, CPNE3, etc., which were reported to be involved in tumor initiation and progression [42–45].

## Discussion

Due to the aggressive and refractory nature of PDAC, the prognosis of patients has been unsatisfactory. Therefore, it is essential to identify effective and powerful prognostic markers for PDAC patients. Herein, we explored the prognostic value of CD58 expression in PDAC patients using diverse public databases and tissue microarray-based IHC. The results suggested that CD58 was enhanced in pancreatitis and PDAC. Upregulated CD58 was strongly associated with poor histological grade and larger tumor size. Cox regression model analysis demonstrated that CD58 was an independent and effective prognostic marker for prognosis of PDAC patients. Furthermore, it was found that CD58 expression may be related to infiltrated immune cells of PDAC tissues.

The initiation and progression of tumor is the result of a series of abnormal gene expressions [46–48]. CD58 is an important adhesion molecule expressed at distinct levels in a variety of normal cells and tumor cells [49]. The costimulatory signaling of CD58 facilitates CTL activation, proliferation, and cytotoxicity [14], while CD58 loss may contribute to a reduction in the recognition and adhesion of T/NK cells to tumor cells in tumor microenvironment [20]. Genomic inactivation of CD58 resulted in the loss of expression, which was an adverse prognostic factor for diffuse large B-cell lymphoma [23]. In vitro studies demonstrated that T/NK-mediated cytotoxicity could be restored by re-expression of wild-type CD58 [20], suggesting the deficiency of CD58 restrains the recognition of tumor cells by T/NK cells and evades immune surveillance in a CD2/CD58-dependent manner. One of the challenges in cancer immunotherapy is the resistance of immune checkpoint blockade (ICB) in the tumor microenvironment. Recently, Frangieh et al. [50] found that CD58 expression was diminished in melanoma tissues from ICB-resistant patients. In cells surviving T/NK co-culture, CD58 level was reduced, which favored resistance to T/NK-cell-mediated killing. Mechanistically, immune evasion caused by CD58 deficiency appeared to be achieved through a different model independent of MHC-mediated antigen presentation. In addition, CD58 knockdown could enhance the expression of co-inhibitory PD-L1 in melanoma cells, which may result in the dysfunction of T cells by interacting with PD-1 on T cells in the tumor microenvironment [50, 51].

Molle et al*.* [8] considered that colorectal cancer possessed a tendency to hypo-express and even abrogate CD58. The CD58 reduction/loss was not linked to tumor stage, grade, and type, thus illustrating that intercellular adhesiveness of colorectal cancer cells in situ was not affected by aberrant cell-surface levels of CD58. In contrast, Xu et al*.* [26] revealed that CD58 was highly expressed in colorectal cancer tissues in comparison to normal intestinal epithelial tissues. It was defined as a new surface marker to facilitate the self-renewal of tumor stem cells in colorectal cancer. Similarly, the results from both public online databases and our cohort demonstrated that CD58 was strongly enhanced in PDAC tissues and could act as an effective prognostic marker for predicting the survival of PDAC patients. Mayer et al*.* [25] found that patients with strong CD58 expression had a shorter survival time than those with low CD58 expression, indicating that CD58 was an adverse prognostic factor in gastric cancer. A high level of CD58 was related to cellular dedifferentiation and dissemination in gastric cancer, while we found that CD58 was relevant in histological grade and tumor size in PDAC. Besides, CD58 expression was significantly related to lymphatic and blood vessel invasion of patients with gastric cancer [25], whereas our data did not show that CD58 expression was linked to lymph node metastasis and macrovascular invasion in PDAC. However, elevated CD58 expression in gastric and colorectal cancer cells was clearly detrimental to immune evasion, so they regarded these findings as “an unexpected direction”. Consistent with these findings, our study also revealed that CD58 expression was upregulated in PDAC tissues, which seems to be beneficial for T/NK cell recognition and killing. The molecular mechanisms involved are complicated and require further investigation. We speculate that this might due to the characteristics of tumor microenvironment, including hypoxia, immunosuppressive state, and fibrosis [52], results in the functional inhibition of infiltrated CTLs.

Notably, it has been reported that PDAC patients with a high degree of CD8^+^ T cell and DCs infiltration possess a better prognosis [53]. However, as a negative prognostic factor, CD58 expression presented a positive correlation with CD8^+^ T cells and DCs, indicating perplexing inconsistency. In fact, different subsets of DCs may have divergent prognostic potential [54]. There is an elevated level of immunotolerant immature DCs was shown to cause shorter survival [55], even an immunosuppressive DC subset that accumulates at secondary sites and facilitates metastasis in PDAC [56]. Therefore, DCs here might contain these subgroups. Regarding CD8^+^ T cells, the correlation of CD58 with CD8^+^ T cells is only based on a propensity result of the database, so the level of evidence is not high. In contrast, the correlation coefficient of CD8^+^ T cells is low and does not rule out the situation caused by statistical bias. Therefore, the results here only indicate that a potential relationship between them, requiring further exploration.

NK cells act as an innate immune barrier to rapidly recognize and kill transformed cells [57]. In tumor microenvironment, TAMs could be domesticated by PDAC cells to promote pancreatic cancer development [58]. Through correlation analysis of CD58, we found that CD58 expression was negatively correlated with NK cell markers but positively correlated with TAM markers, which explains at least partly why PDAC patients with high CD58 expression have a poor prognosis. Moreover, under pathological conditions of tumorigenesis, CD58 expression might also be involved in the process of EMT and CSC of PDAC and promote PDAC progression. More importantly, functional enrichment analysis suggested that, in addition to adhesion function, CD58 is likely to be implicated in intracellular signal transduction, the regulation of cytoskeleton, and enzyme-related activities. The KEGG pathway strongly suggested that CD58 is involved in pancreatic cancer progression.

There are some limitations in the present study. (i) Our collection of pancreatic cancer tissue samples was limited and may cause sample bias. The expansion of sample size is an essential step for our future investigation. (ii) The postoperative follow-up time of the study cohort was relatively short and may have been subjected to potential selection bias. (iii) This study is based on a single-center retrospective design, so the results need to be verified by the multi-center prospective studies. (iv) The function of CD58 and the related molecular mechanisms in PDAC remain to be further illustrated.

## Conclusion

In summary, the present study reveals that CD58 expression is upregulated in PDAC cancer tissues, which is associated with worse histological grade and larger tumor size and predicts a poor prognosis in PDAC patients. These findings indicate that CD58 can be served as an effective prognostic marker for PDAC.

## Supplementary Information

**Additional file 1: Figure S1.** The relationship between CD58 and immune-related, EMT-related, and CSC-related genes in TIMER.

**Additional file 2: Figure S2.** The relationship between CD58 and immune-related, EMT-related, and CSC-related genes in GEPIA.

**Additional file 3: Table S1.** Genes positively and negatively correlated with CD58 expression.

**Additional file 4: Table S2.** CD58 functional enrichment analysis and KEGG pathway analysis.

## Data Availability

The public datasets of PDAC in this study can be found in NCBI (https://www.ncbi.nlm.nih.gov/gene/), Oncomine (http://www.oncomine.org), GEPIA (http://gepia.cancer-pku.cn/), OncoLnc (http://www.oncolnc.org), SurvExpress (http://bioinformatica.mty.itesm.mx/SurvExpress), The Kaplan–Meier plotter (http://kmplot.com/analysis/), TIMER (https://cistrome.shinyapps.io/timer), LinkedOmics (http://www.linkedomics.org/), DAVID (https://david.ncifcrf.gov/) and geneMANIA (http://genemania.org/).

## Abbreviations

CBI-CCA: Cadherin binding involved in cell–cell adhesion
CI: Confidence interval
CSC: Cancer stem cell
CTL: Cytotoxic T lymphocyte
DAVID: Database for Annotation: Visualization and Integrated Discovery
DC: Dendritic cell
DEGs: Differentially expressed genes
DFS: Disease-free survival
ECPM: Extrinsic component of plasma membrane
EMT: Epithelial–mesenchymal transition
EPLPM: Establishment of protein localization to plasma membrane
GEPIA: Gene Expression Profiling Interactive Analysis
GO: Gene ontology
HR: Hazard ratio
ICB: Immune checkpoint blockade
IHC: Immunohistochemistry
KEGG: Kyoto encyclopedia of genes and genomes
NCBI: National Center for Biotechnology Information
NK cell: Natural killer cell
NRAP: Negative regulation of apoptotic process
OS: Overall survival
PAAD: Pancreatic adenocarcinoma
PDAC: Pancreatic ductal adenocarcinoma
TAM: Tumor-associated macrophage
TIME: Tumor IMmune Estimation Resource

## References

[1] Kamisawa T, Wood LD, Itoi T, Takaori K (2016). Pancreatic cancer. Lancet.

[2] Rahib L, Smith BD, Aizenberg R, Rosenzweig AB, Fleshman JM, Matrisian LM (2014). Projecting cancer incidence and deaths to 2030: the unexpected burden of thyroid, liver, and pancreas cancers in the United States. Cancer Res.

[3] Christenson ES, Jaffee E, Azad NS (2020). Current and emerging therapies for patients with advanced pancreatic ductal adenocarcinoma: a bright future. Lancet Oncol.

[4] Bray F, Ferlay J, Soerjomataram I, Siegel RL, Torre LA, Jemal A (2018). Global cancer statistics 2018: GLOBOCAN estimates of incidence and mortality worldwide for 36 cancers in 185 countries. CA Cancer J Clin.

[5] Leonhardt CS, Traub B, Hackert T, Klaiber U, Strobel O, Büchler MW (2020). Adjuvant and neoadjuvant chemotherapy in pancreatic ductal adenocarcinoma. J Pancreatol.

[6] Maeda S, Unno M, Yu J (2019). Adjuvant and neoadjuvant therapy for pancreatic cancer. J Pancreatol.

[7] Tian X, Li J, Gao H, Zhuang Y, Ma Y, Chen Y (2019). Prognostic factors for disease-free survival in patients with pancreatic ductal adenocarcinoma after surgery: a single center experience. J Pancreatol.

[8] Moller P, Koretz K, Schlag P, Momburg F (1991). Frequency of abnormal expression of HLA-A, B, C and HLA-DR molecules, invariant chain, and LFA-3 (CD58) in colorectal carcinoma and its impact on tumor recurrence. Int J Cancer Suppl.

[9] Dustin ML, Selvaraj P, Mattaliano RJ, Springer TA (1987). Anchoring mechanisms for LFA-3 cell adhesion glycoprotein at membrane surface. Nature.

[10] Krensky AM, Sanchez-Madrid F, Robbins E, Nagy JA, Springer TA, Burakoff SJ (1983). The functional significance, distribution, and structure of LFA-1, LFA-2, and LFA-3: cell surface antigens associated with CTL-target interactions. J Immunol.

[11] Kanner SB, Damle NK, Blake J, Aruffo A, Ledbetter JA (1992). CD2/LFA-3 ligation induces phospholipase-C gamma 1 tyrosine phosphorylation and regulates CD3 signaling. J Immunol.

[12] Wang JH, Smolyar A, Tan K, Liu JH, Kim M, Sun ZY (1999). Structure of a heterophilic adhesion complex between the human CD2 and CD58 (LFA-3) counterreceptors. Cell.

[13] Shaw S, Luce GE, Quinones R, Gress RE, Springer TA, Sanders ME (1986). Two antigen-independent adhesion pathways used by human cytotoxic T-cell clones. Nature.

[14] Schirren CA, Volpel H, Meuer SC (1992). Adhesion molecules on freshly recovered T leukemias promote tumor-directed lympholysis. Blood.

[15] Altomonte M, Gloghini A, Bertola G, Gasparollo A, Carbone A, Ferrone S (1993). Differential expression of cell adhesion molecules CD54/CD11a and CD58/CD2 by human melanoma cells and functional role in their interaction with cytotoxic cells. Cancer Res.

[16] Sanchez-Madrid F, Krensky AM, Ware CF, Robbins E, Strominger JL, Burakoff SJ (1982). Three distinct antigens associated with human T-lymphocyte-mediated cytolysis: LFA-1, LFA-2, and LFA-3. Proc Natl Acad Sci USA.

[17] Smith ME, Marsh SG, Bodmer JG, Gelsthorpe K, Bodmer WF (1989). Loss of HLA-A, B, C allele products and lymphocyte function-associated antigen 3 in colorectal neoplasia. Proc Natl Acad Sci USA.

[18] Nouri AM, Smith ME, Crosby D, Oliver RT (1990). Selective and non-selective loss of immunoregulatory molecules (HLA-A, B, C antigens and LFA-3) in transitional cell carcinoma. Br J Cancer.

[19] Billaud M, Rousset F, Calender A, Cordier M, Aubry JP, Laisse V (1990). Low expression of lymphocyte function-associated antigen (LFA)-1 and LFA-3 adhesion molecules is a common trait in Burkitt's lymphoma associated with and not associated with Epstein-Barr virus. Blood.

[20] Challa-Malladi M, Lieu YK, Califano O, Holmes AB, Bhagat G, Murty VV (2011). Combined genetic inactivation of beta2-Microglobulin and CD58 reveals frequent escape from immune recognition in diffuse large B cell lymphoma. Cancer Cell.

[21] Upadhyaya G, Guba SC, Sih SA, Feinberg AP, Talpaz M, Kantarjian HM (1991). Interferon-alpha restores the deficient expression of the cytoadhesion molecule lymphocyte function antigen-3 by chronic myelogenous leukemia progenitor cells. J Clin Invest.

[22] Otsuka Y, Nishikori M, Arima H, Izumi K, Kitawaki T, Hishizawa M (2020). EZH2 inhibitors restore epigenetically silenced CD58 expression in B-cell lymphomas. Mol Immunol.

[23] Cao Y, Zhu T, Zhang P, Xiao M, Yi S, Yang Y (2016). Mutations or copy number losses of CD58 and TP53 genes in diffuse large B cell lymphoma are independent unfavorable prognostic factors. Oncotarget.

[24] Li XM, Zhang LP, Wang YZ, Lu AD, Chang Y, Zhu HH (2016). CD38+ CD58- is an independent adverse prognostic factor in paediatric Philadelphia chromosome negative B cell acute lymphoblastic leukaemia patients. Leuk Res.

[25] Mayer B, Lorenz C, Babic R, Jauch KW, Schildberg FW, Funke I (1995). Expression of leukocyte cell adhesion molecules on gastric carcinomas: possible involvement of LFA-3 expression in the development of distant metastases. Int J Cancer.

[26] Xu S, Wen Z, Jiang Q, Zhu L, Feng S, Zhao Y (2015). CD58, a novel surface marker, promotes self-renewal of tumor-initiating cells in colorectal cancer. Oncogene.

[27] Kuppner MC, Hamou MF, de Tribolet N (1990). Activation and adhesion molecule expression on lymphoid infiltrates in human glioblastomas. J Neuroimmunol.

[28] Niu Z, Wang M, Zhou L, Yao L, Liao Q, Zhao Y (2015). Elevated GRP78 expression is associated with poor prognosis in patients with pancreatic cancer. Sci Rep.

[29] Cui M, You L, Zheng B, Huang X, Liu Q, Huang J (2020). High expression of cancer-derived glycosylated immunoglobulin G predicts poor prognosis in pancreatic ductal adenocarcinoma. J Cancer.

[30] Shen J, Cao B, Wang Y, Ma C, Zeng Z, Liu L (2018). Hippo component YAP promotes focal adhesion and tumour aggressiveness via transcriptionally activating THBS1/FAK signalling in breast cancer. J Exp Clin Cancer Res.

[31] Tang Z, Li C, Kang B, Gao G, Li C, Zhang Z (2017). GEPIA: a web server for cancer and normal gene expression profiling and interactive analyses. Nucleic Acids Res.

[32] Nagy A, Lanczky A, Menyhart O, Gyorffy B (2018). Validation of miRNA prognostic power in hepatocellular carcinoma using expression data of independent datasets. Sci Rep.

[33] Fagerberg L, Hallstrom BM, Oksvold P, Kampf C, Djureinovic D, Odeberg J (2014). Analysis of the human tissue-specific expression by genome-wide integration of transcriptomics and antibody-based proteomics. Mol Cell Proteomics.

[34] Aguirre-Gamboa R, Gomez-Rueda H, Martinez-Ledesma E, Martinez-Torteya A, Chacolla-Huaringa R, Rodriguez-Barrientos A (2013). SurvExpress: an online biomarker validation tool and database for cancer gene expression data using survival analysis. PLoS ONE.

[35] Rhodes DR, Yu J, Shanker K, Deshpande N, Varambally R, Ghosh D (2004). ONCOMINE: a cancer microarray database and integrated data-mining platform. Neoplasia.

[36] Li T, Fan J, Wang B, Traugh N, Chen Q, Liu JS (2017). TIMER: a web server for comprehensive analysis of tumor-infiltrating immune cells. Cancer Res.

[37] Vasaikar SV, Straub P, Wang J, Zhang B (2018). LinkedOmics: analyzing multi-omics data within and across 32 cancer types. Nucleic Acids Res.

[38] Jiao X, Sherman BT, da Huang W, Stephens R, Baseler MW, Lane HC (2012). DAVID-WS: a stateful web service to facilitate gene/protein list analysis. Bioinformatics.

[39] Kanehisa M, Goto S (2000). KEGG: kyoto encyclopedia of genes and genomes. Nucleic Acids Res.

[40] Ashburner M, Ball CA, Blake JA, Botstein D, Butler H, Cherry JM (2000). Gene ontology: tool for the unification of biology. The Gene Ontology Consortium. Nat Genet.

[41] Warde-Farley D, Donaldson SL, Comes O, Zuberi K, Badrawi R, Chao P (2010). The GeneMANIA prediction server: biological network integration for gene prioritization and predicting gene function. Nucleic Acids Res.

[42] Segara D, Biankin AV, Kench JG, Langusch CC, Dawson AC, Skalicky DA (2005). Expression of HOXB2, a retinoic acid signaling target in pancreatic cancer and pancreatic intraepithelial neoplasia. Clin Cancer Res.

[43] Choi MC, Jong HS, Kim TY, Song SH, Lee DS, Lee JW (2004). AKAP12/Gravin is inactivated by epigenetic mechanism in human gastric carcinoma and shows growth suppressor activity. Oncogene.

[44] Lu J, Dong Q, Zhang B, Wang X, Ye B, Zhang F (2015). Chloride intracellular channel 1 (CLIC1) is activated and functions as an oncogene in pancreatic cancer. Med Oncol.

[45] Lin H, Zhang X, Liao L, Yu T, Li J, Pan H (2018). CPNE3 promotes migration and invasion in non-small cell lung cancer by interacting with RACK1 via FAK signaling activation. J Cancer.

[46] Zhang Y, Zhang R, Luo G, Ai K (2018). Long noncoding RNA SNHG1 promotes cell proliferation through PI3K/AKT signaling pathway in pancreatic ductal adenocarcinoma. J Cancer.

[47] Zhang Y, Zhang R, Ding X, Ai K (2018). EFNB2 acts as the target of miR-557 to facilitate cell proliferation, migration and invasion in pancreatic ductal adenocarcinoma by bioinformatics analysis and verification. Am J Transl Res.

[48] Waddell N, Pajic M, Patch AM, Chang DK, Kassahn KS, Bailey P (2015). Whole genomes redefine the mutational landscape of pancreatic cancer. Nature.

[49] Seed B (1987). An LFA-3 cDNA encodes a phospholipid-linked membrane protein homologous to its receptor CD2. Nature.

[50] Frangieh CJ, Melms JC, Thakore PI, Geiger-Schuller KR, Ho P, Luoma AM (2021). Multimodal pooled Perturb-CITE-seq screens in patient models define mechanisms of cancer immune evasion. Nat Genet.

[51] Zhang J, Dang F, Ren J, Wei W (2018). Biochemical aspects of PD-L1 regulation in cancer immunotherapy. Trends Biochem Sci.

[52] Zhang Y, Liu Q, Liao Q (2020). Long noncoding RNA: a dazzling dancer in tumor immune microenvironment. J Exp Clin Cancer Res.

[53] Ino Y, Yamazaki-Itoh R, Shimada K, Iwasaki M, Kosuge T, Kanai Y (2013). Immune cell infiltration as an indicator of the immune microenvironment of pancreatic cancer. Br J Cancer.

[54] Deicher A, Andersson R, Tingstedt B, Lindell G, Bauden M, Ansari D (2018). Targeting dendritic cells in pancreatic ductal adenocarcinoma. Cancer Cell Int.

[55] Lundgren S, Karnevi E, Elebro J, Nodin B, Karlsson MCI, Eberhard J (2017). The clinical importance of tumour-infiltrating macrophages and dendritic cells in periampullary adenocarcinoma differs by morphological subtype. J Transl Med.

[56] Kenkel JA, Tseng WW, Davidson MG, Tolentino LL, Choi O, Bhattacharya N (2017). An immunosuppressive dendritic cell subset accumulates at secondary sites and promotes metastasis in pancreatic cancer. Cancer Res.

[57] Chiossone L, Dumas PY, Vienne M, Vivier E (2018). Natural killer cells and other innate lymphoid cells in cancer. Nat Rev Immunol.

[58] Zhang R, Liu Q, Peng J, Wang M, Gao X, Liao Q (2019). Pancreatic cancer-educated macrophages protect cancer cells from complement-dependent cytotoxicity by up-regulation of CD59. Cell Death Dis.

