# Supplementary figures and images for: Upregulated CD58 is associated with clinicopathological characteristics and poor prognosis of patients with pancreatic ductal adenocarcinoma
Source: Cancer Cell Int. 2021 Jun 30;21:327. doi: 10.1186/s12935-021-02037-0 (PMC8243423; doi:10.1186/s12935-021-02037-0)

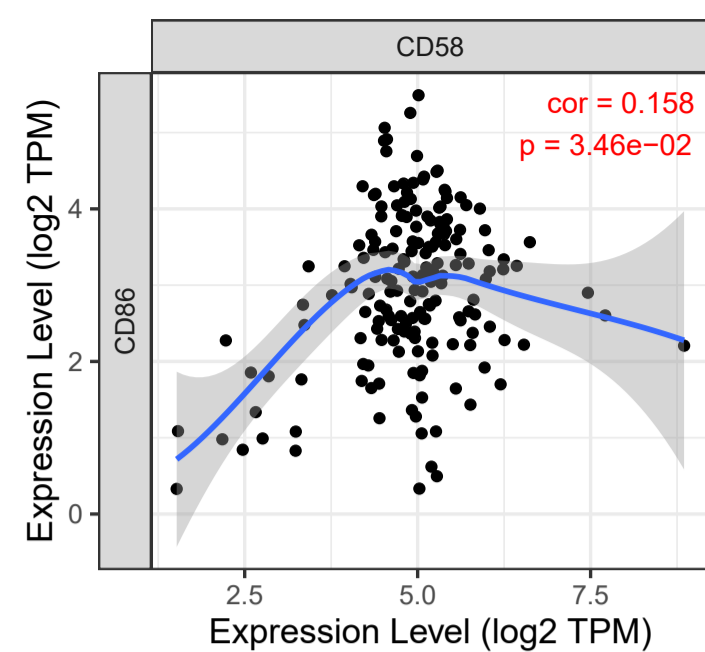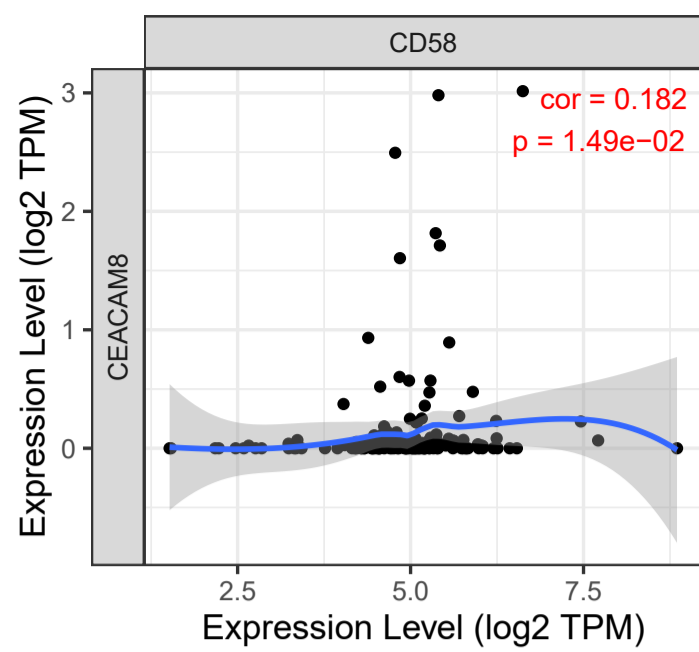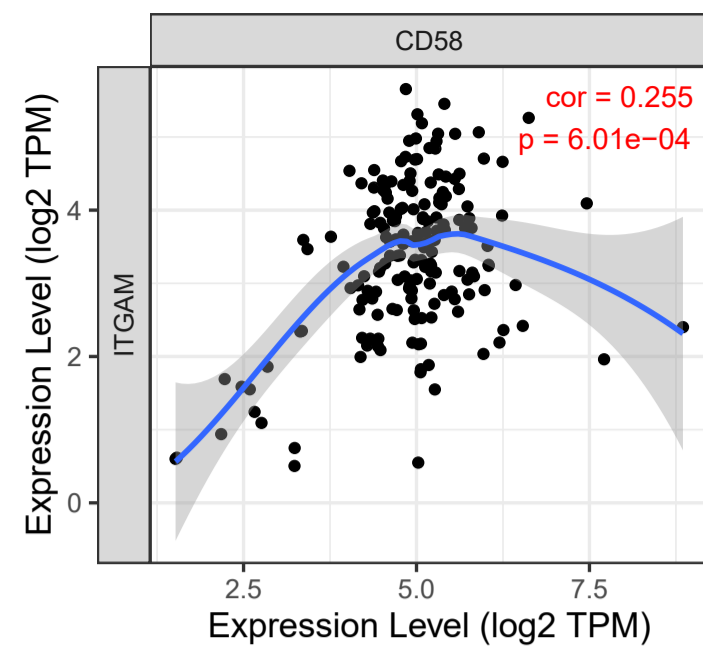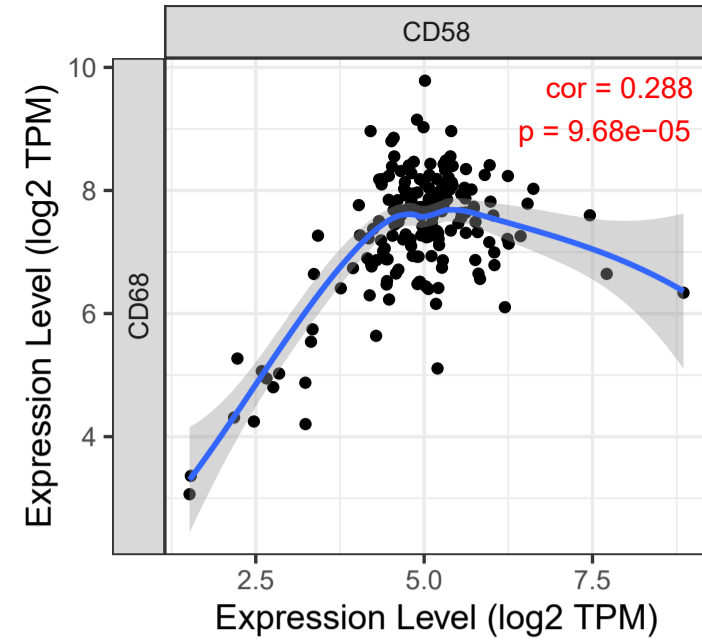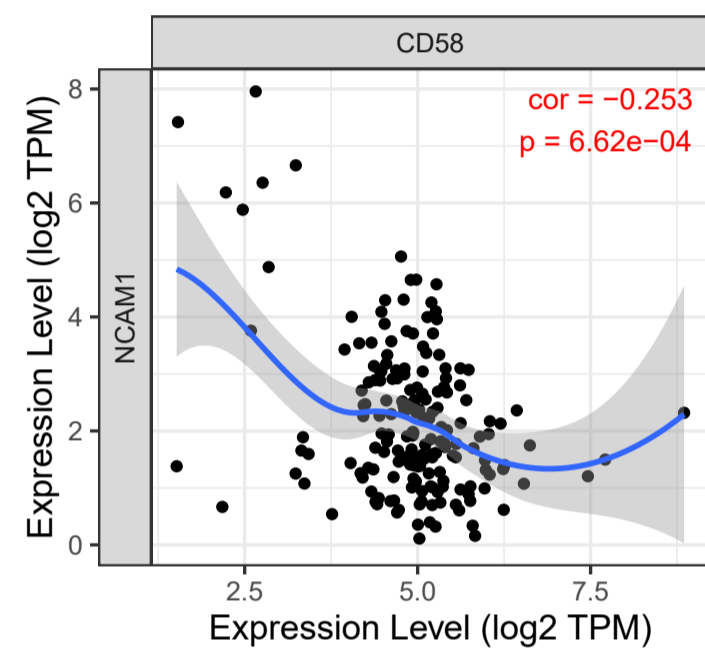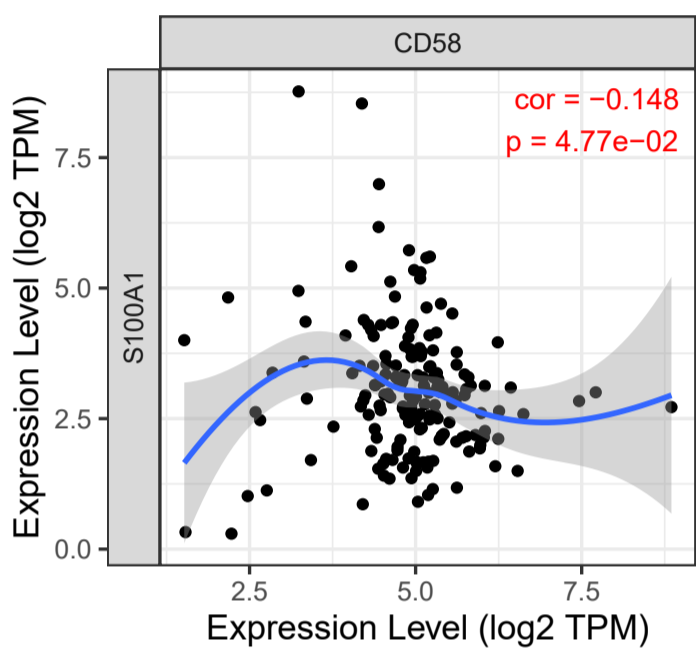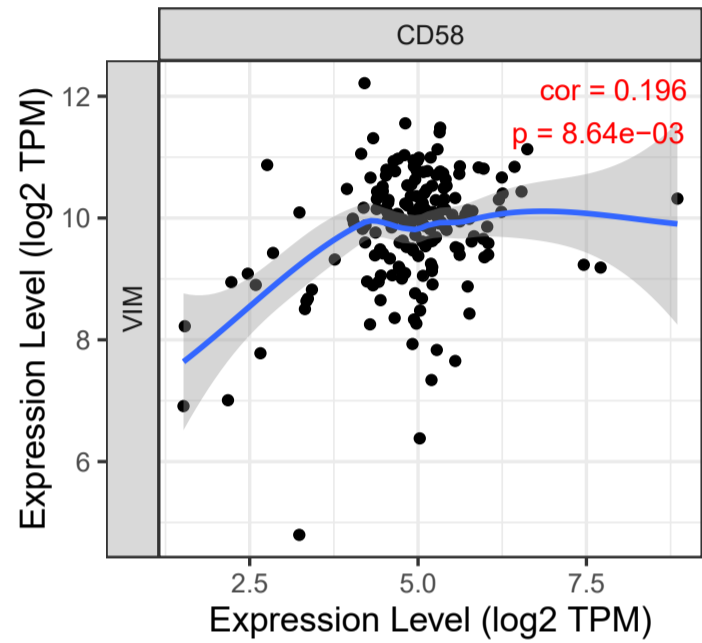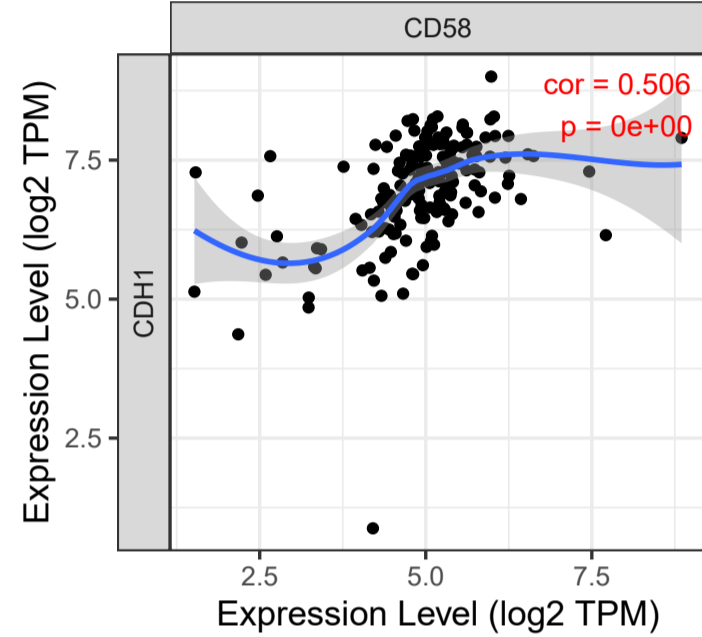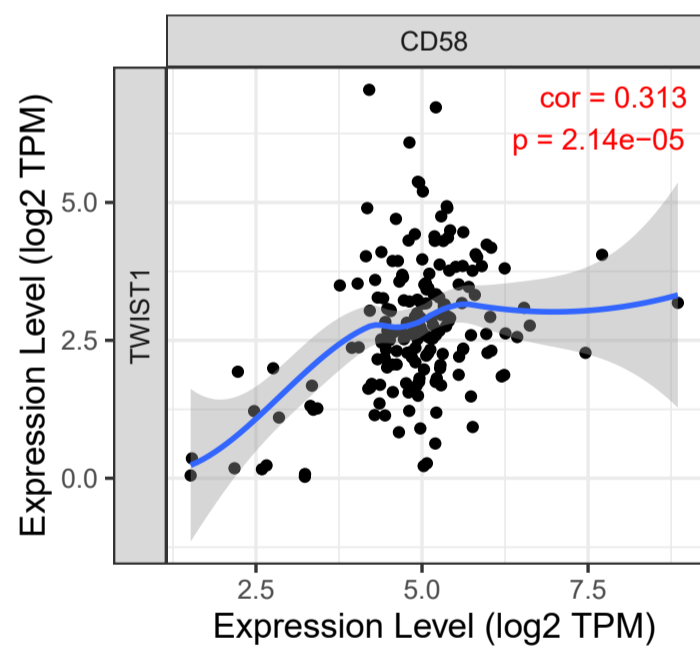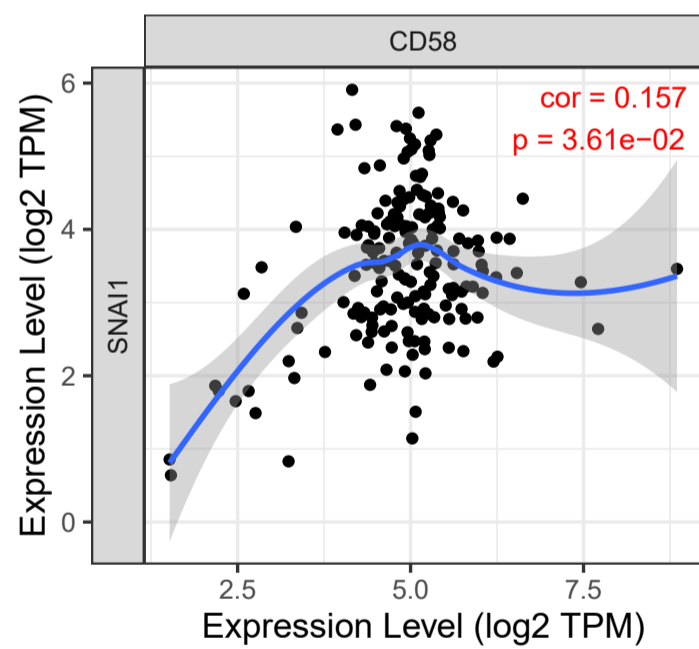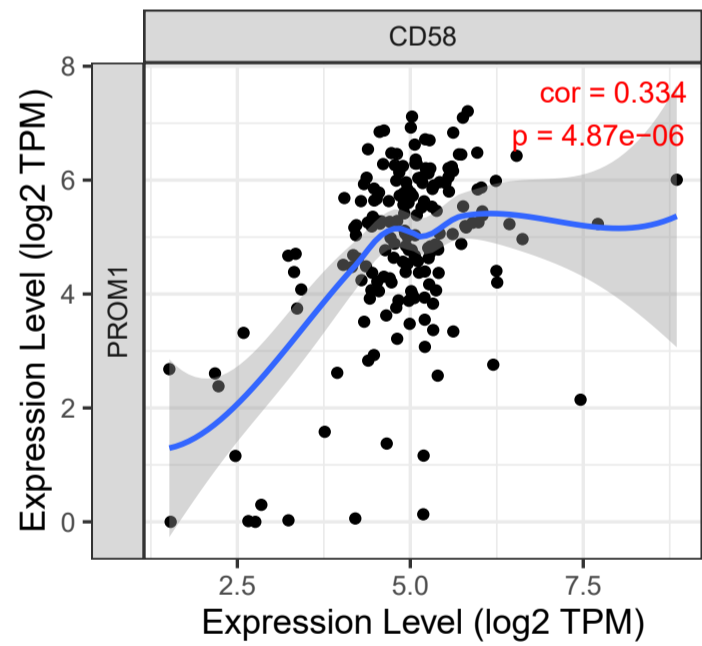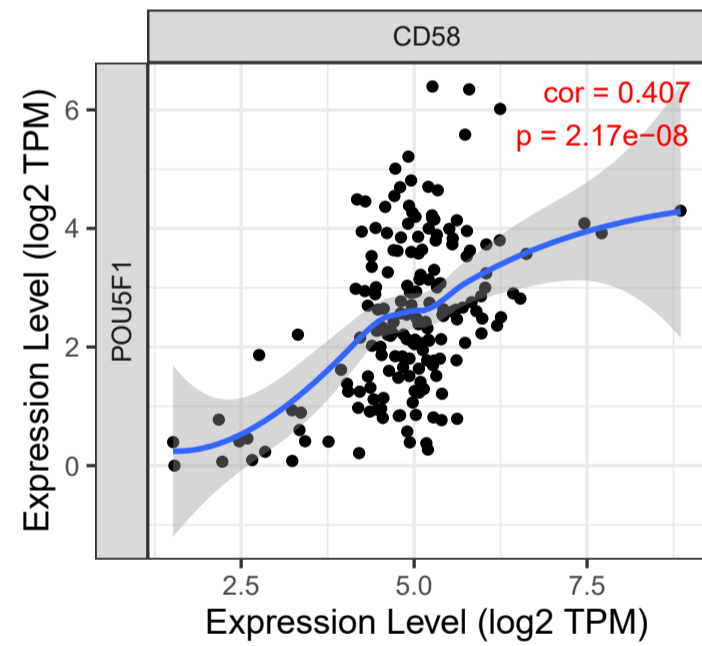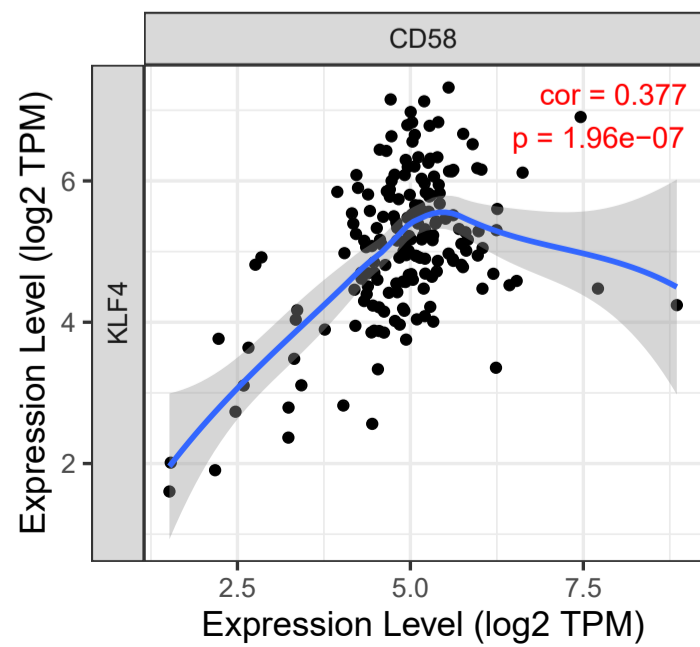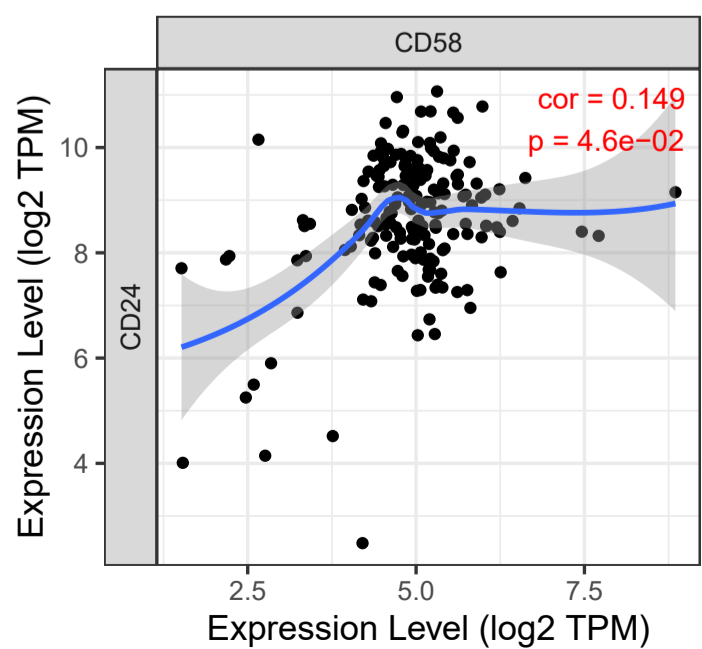

Supplement: Supplementary file 1 — Additional file 1: Figure S1. The relationship between CD58 and immune-related, EMT-related, and CSC-related genes in TIMER. [file 12935_2021_2037_MOESM1_ESM.pdf]

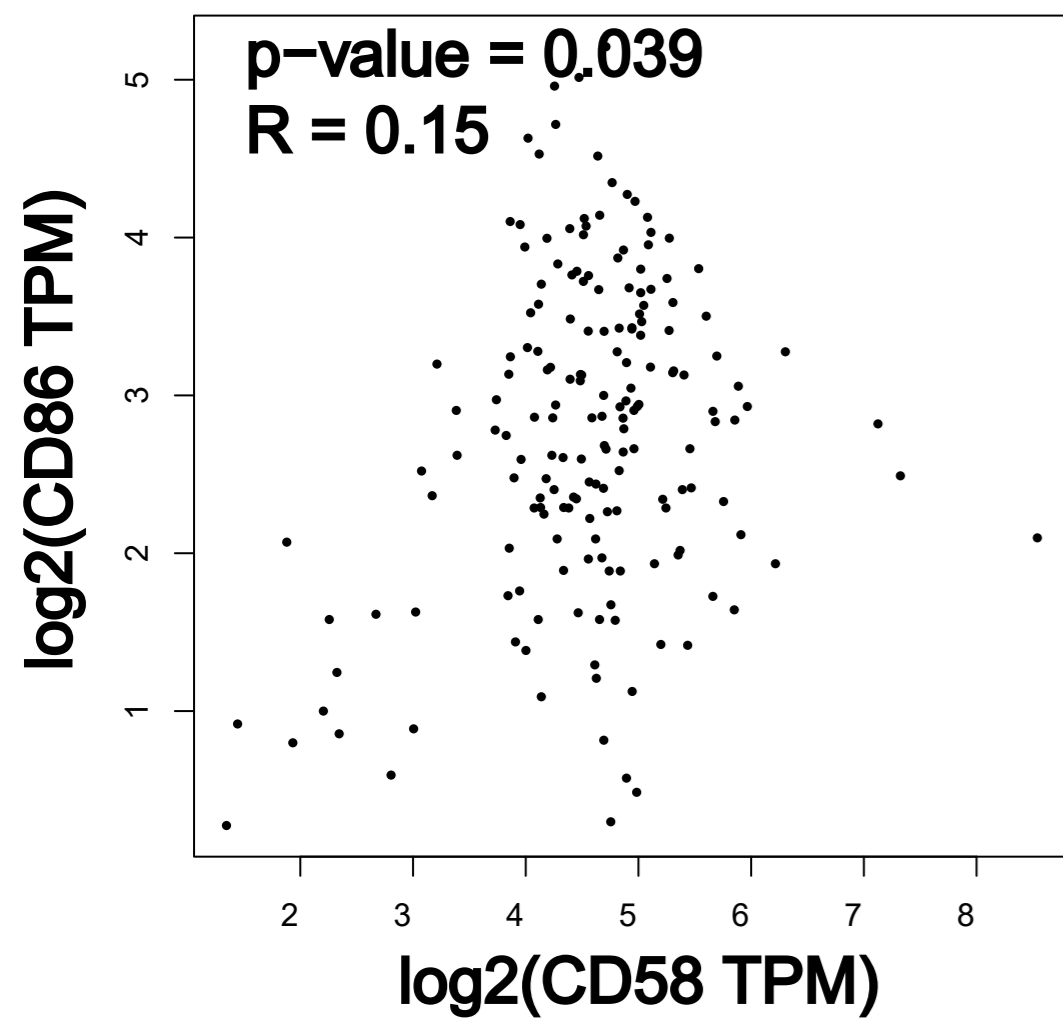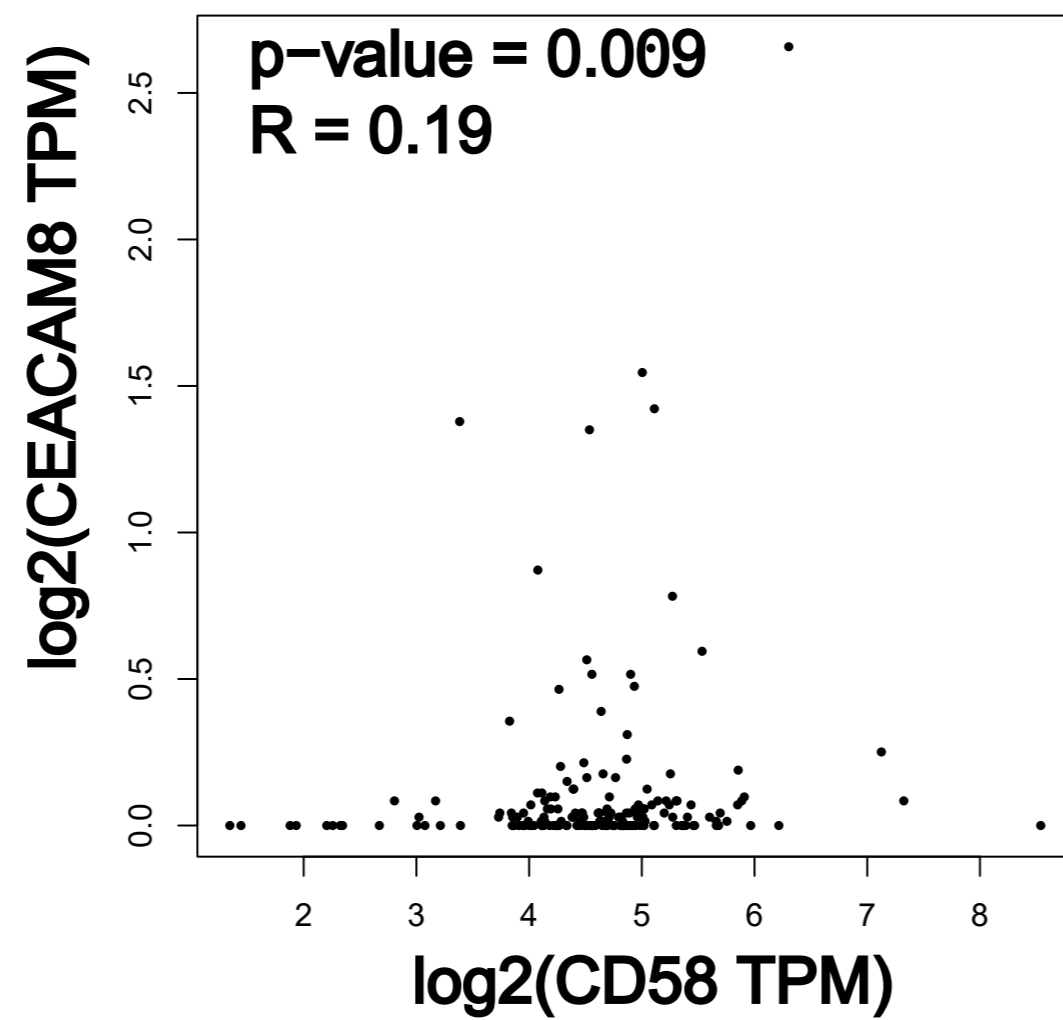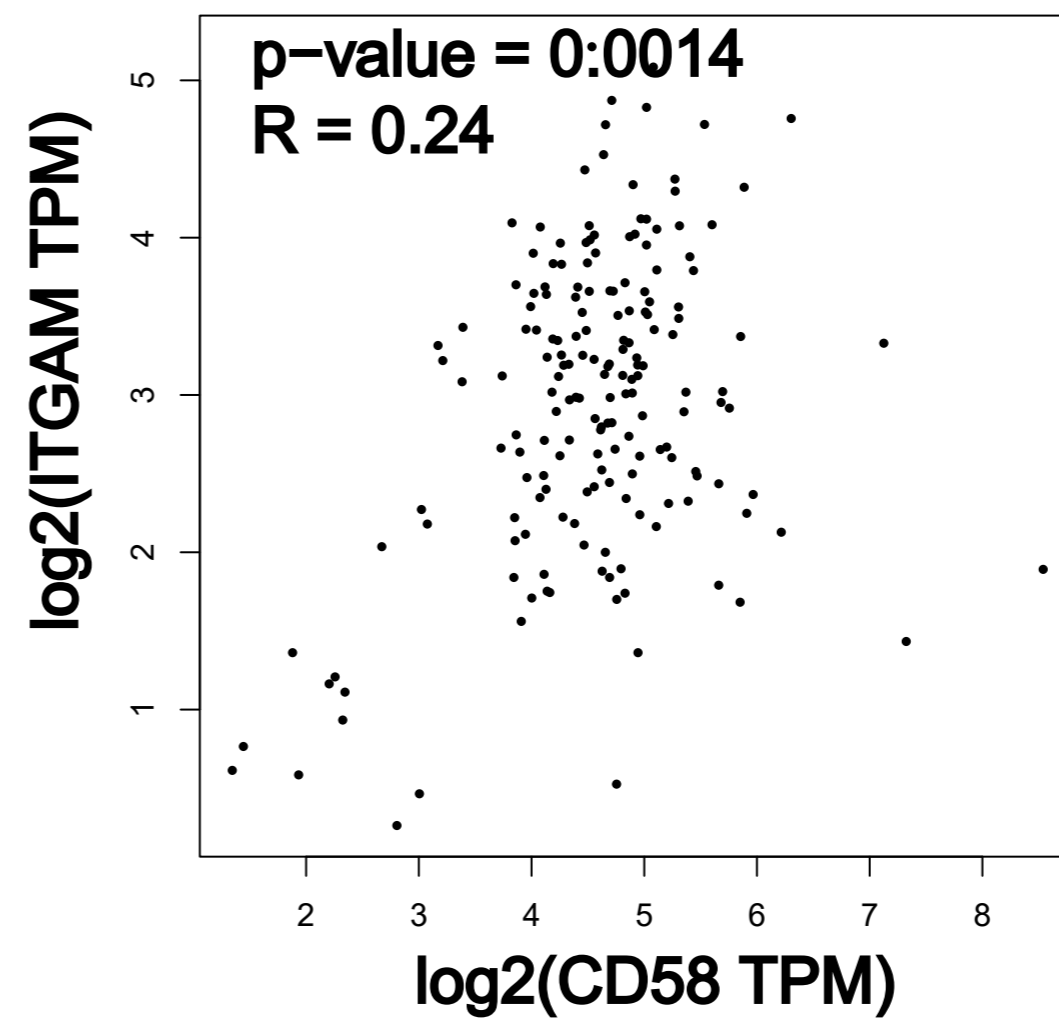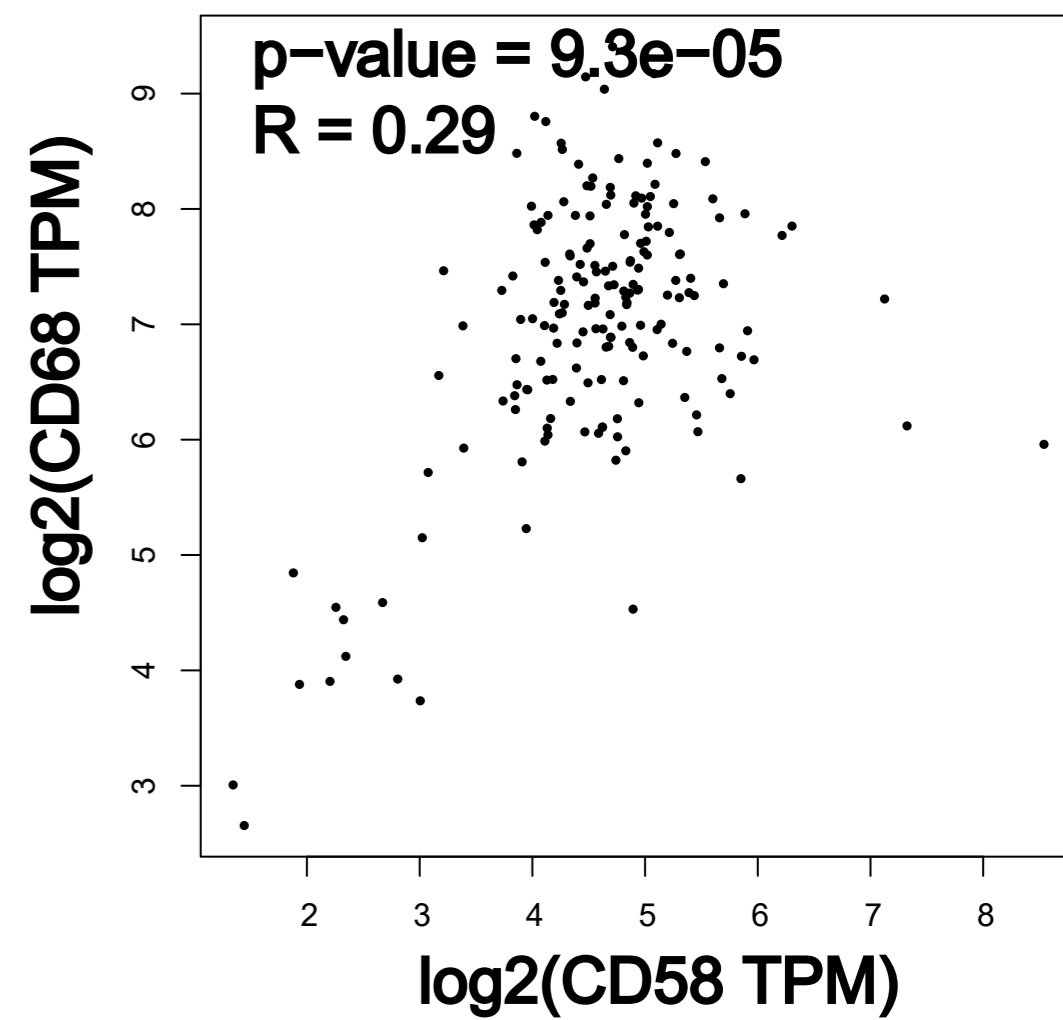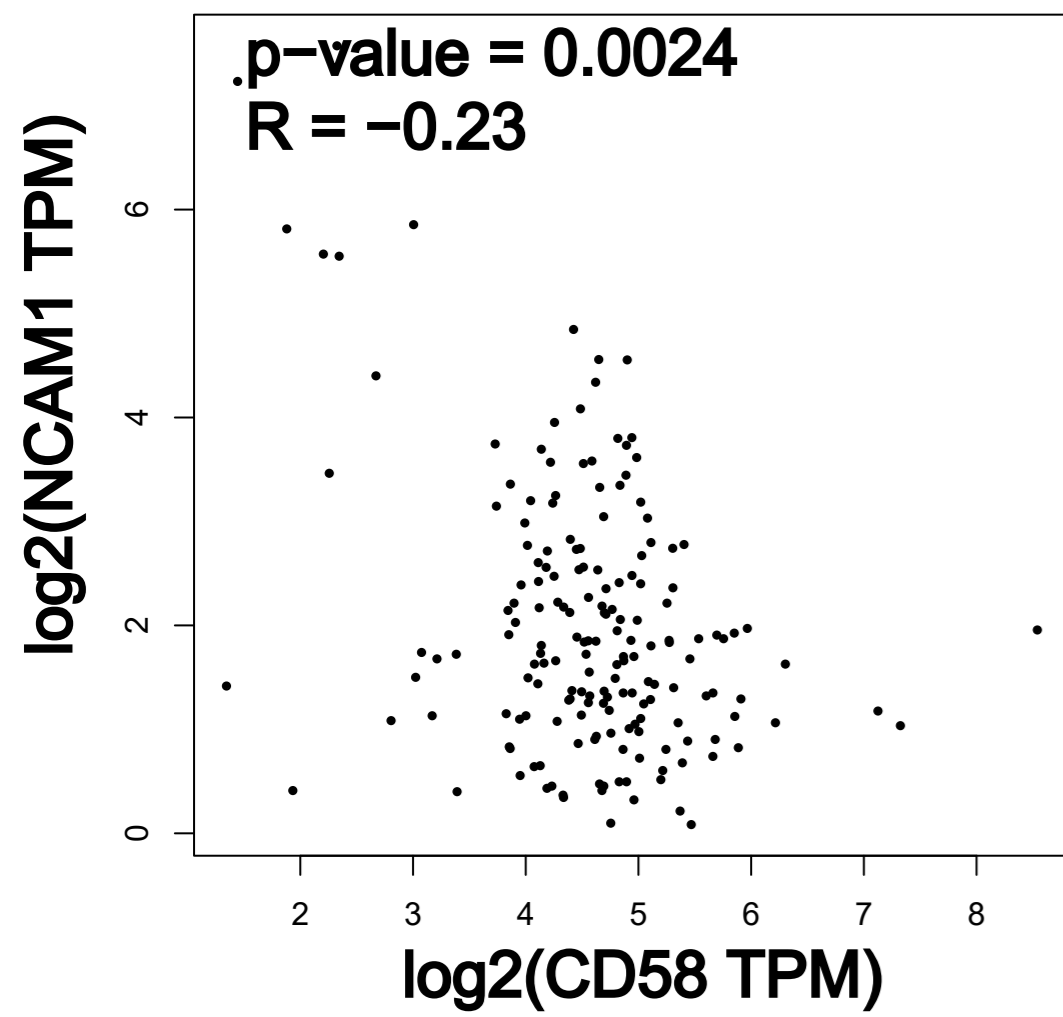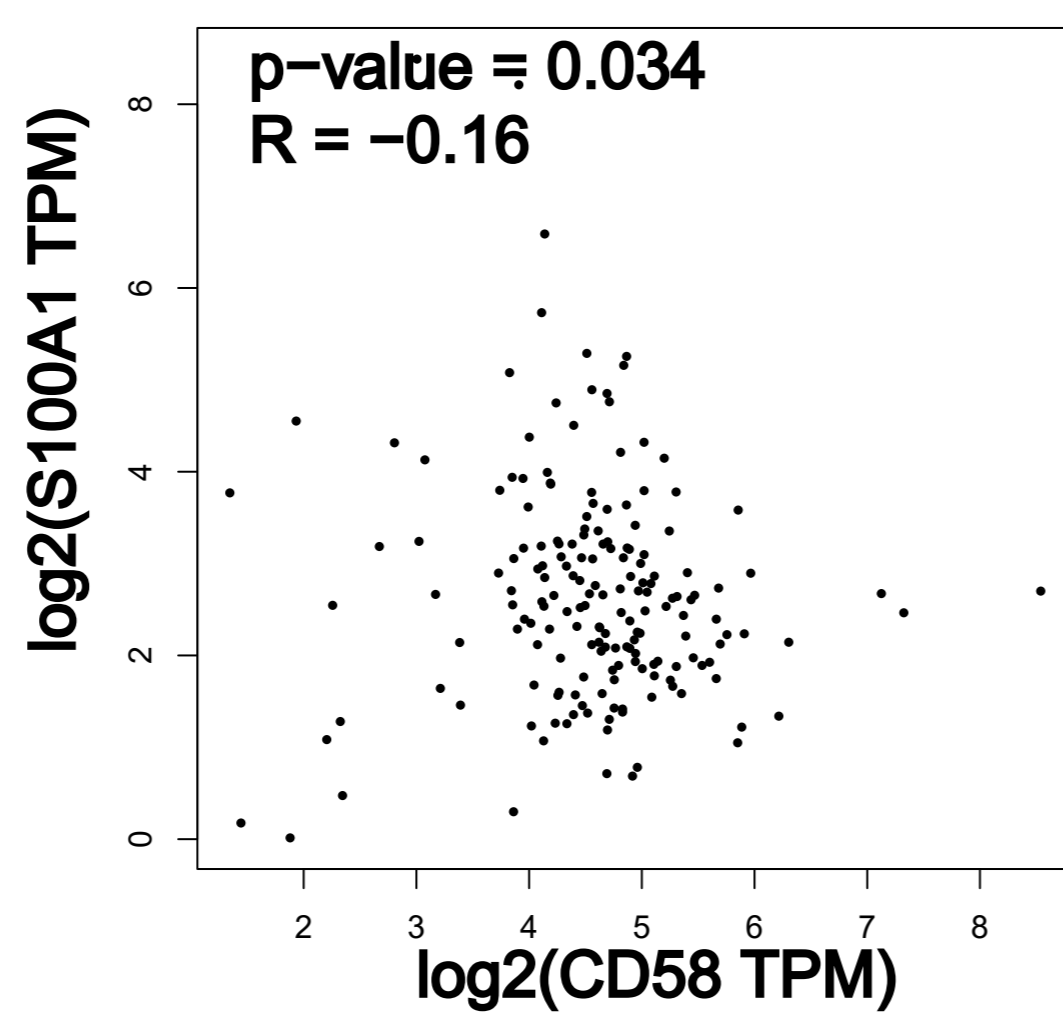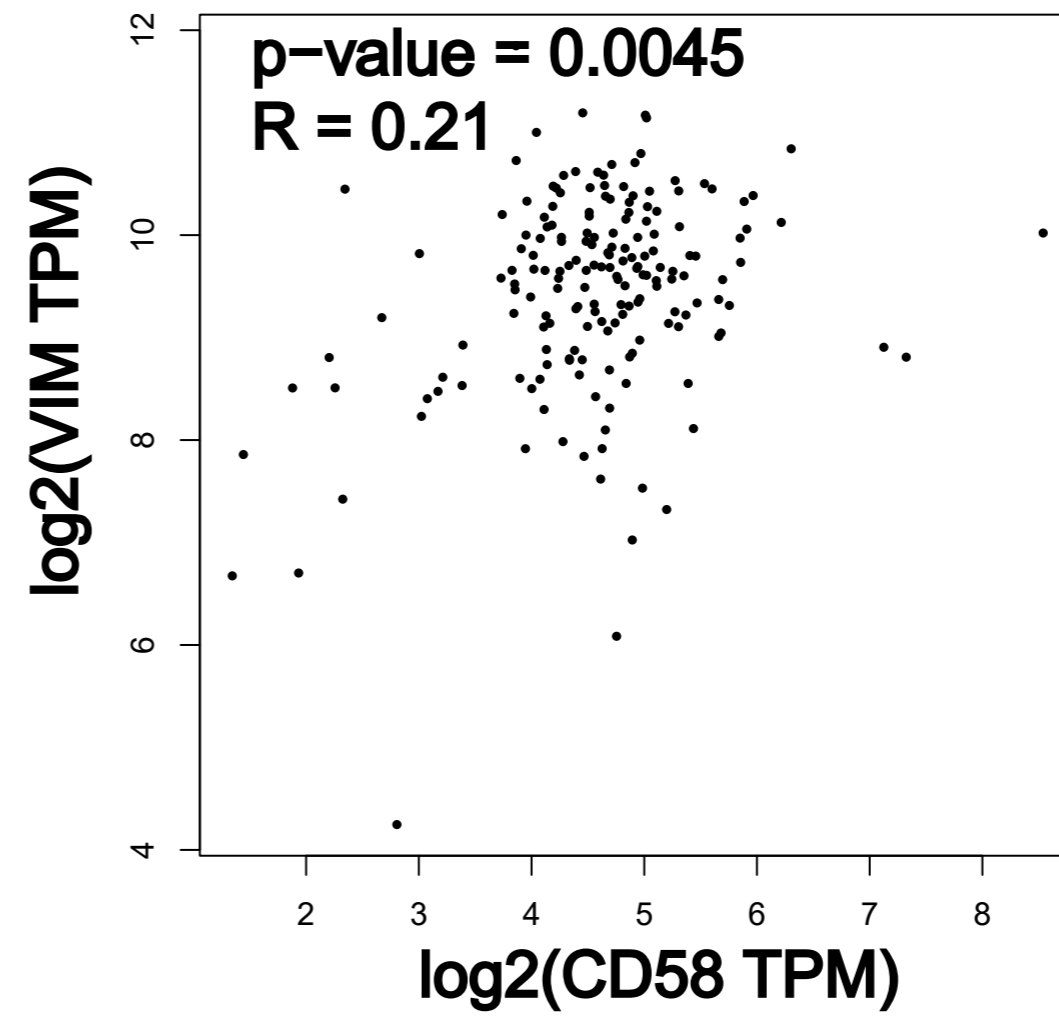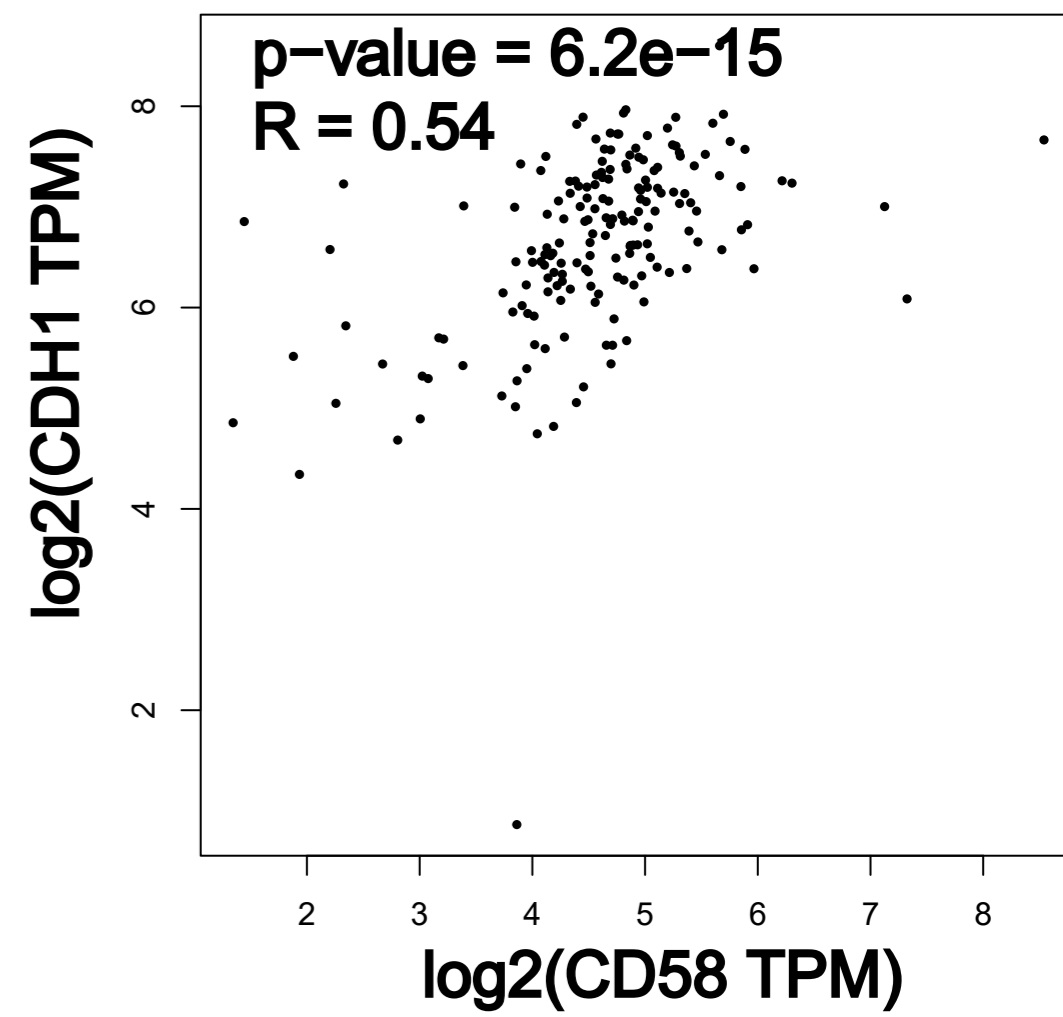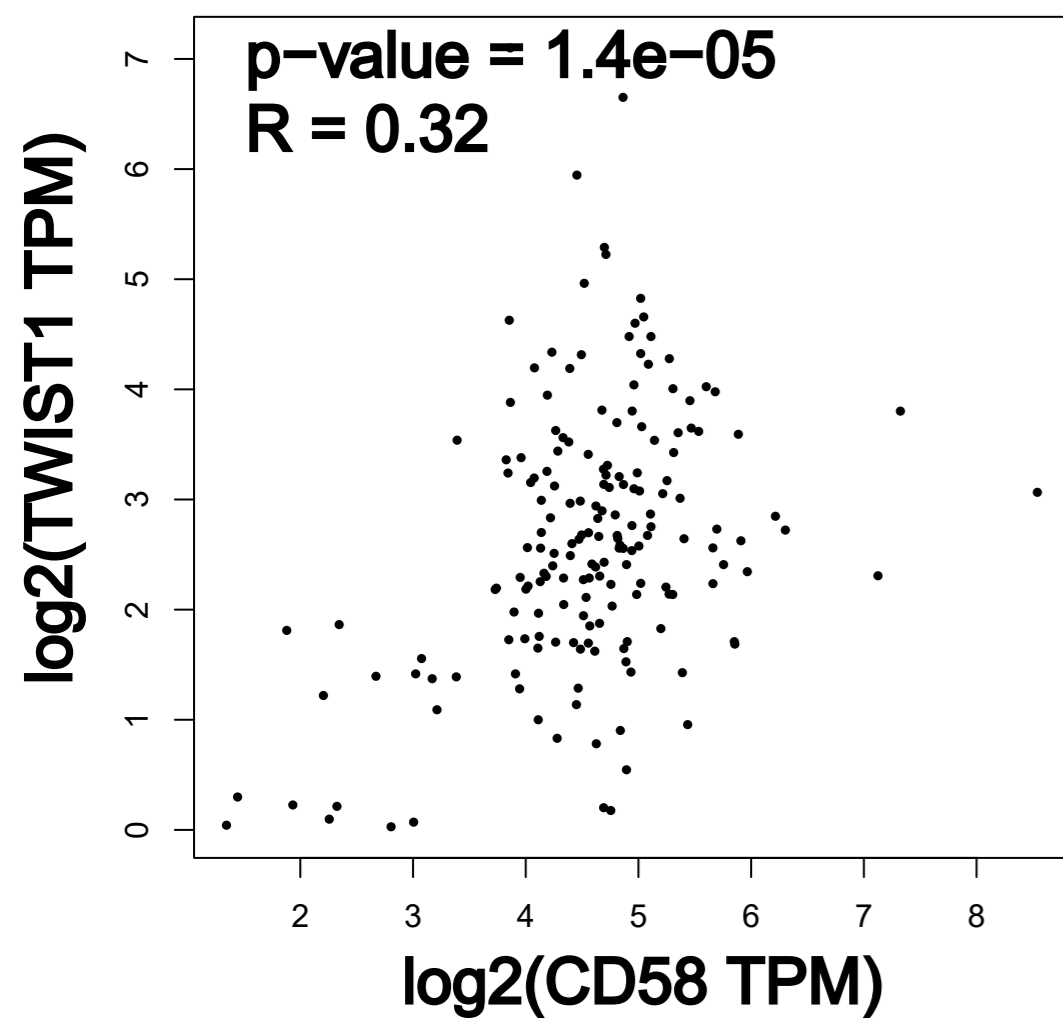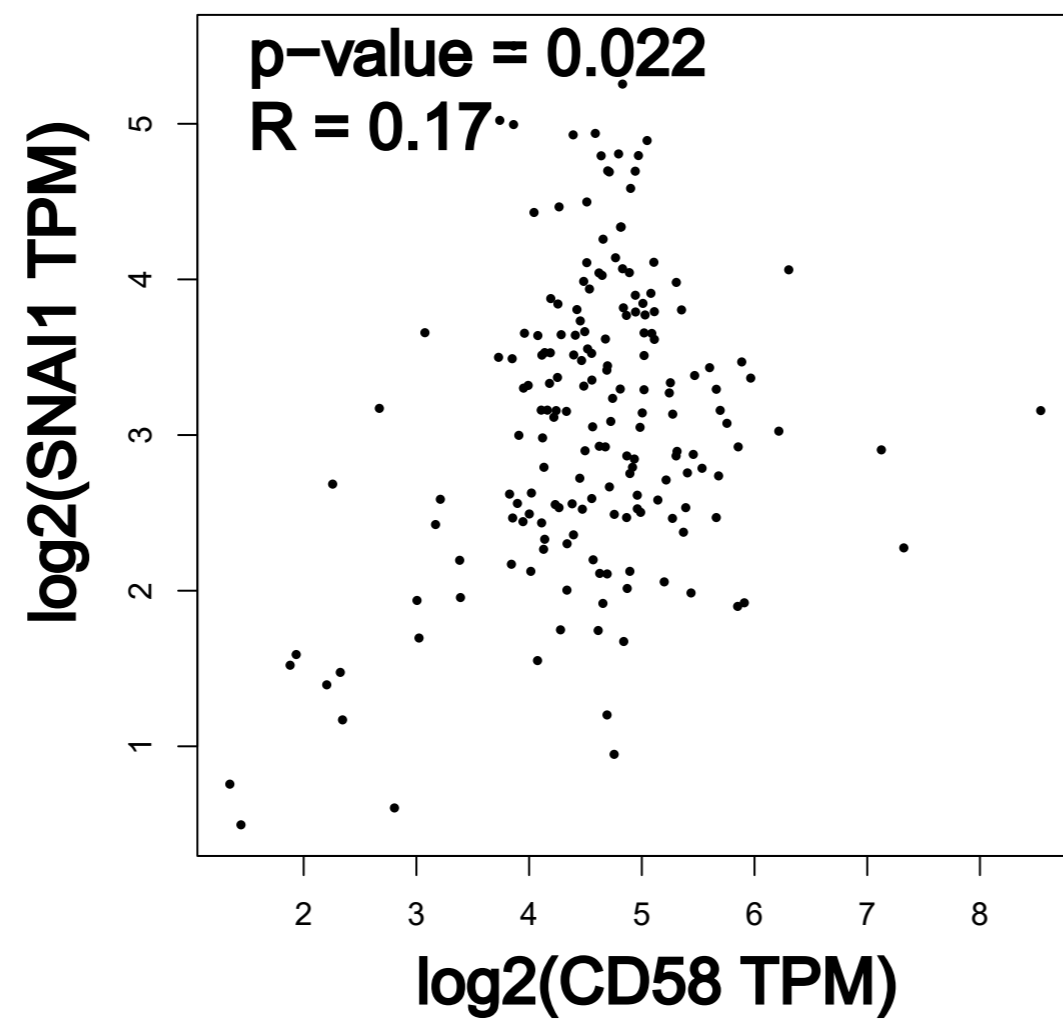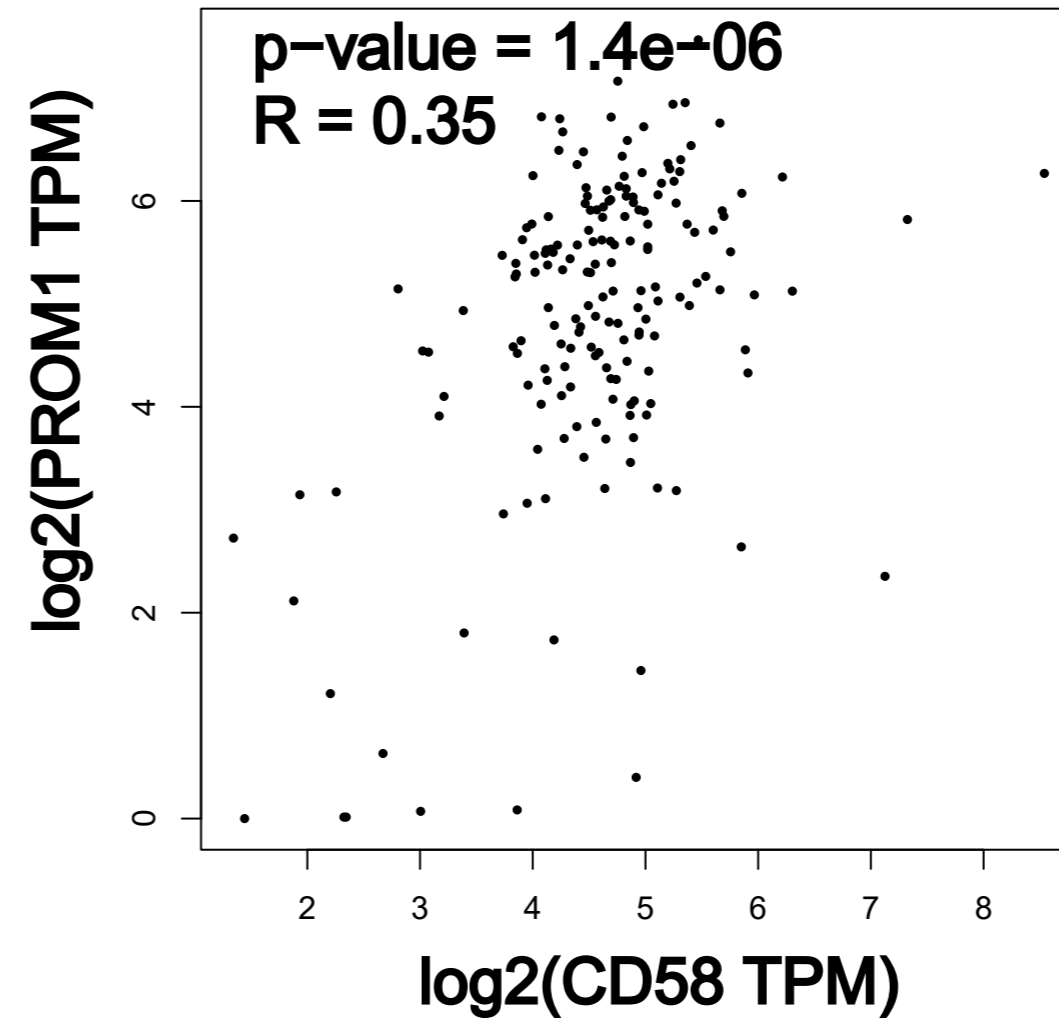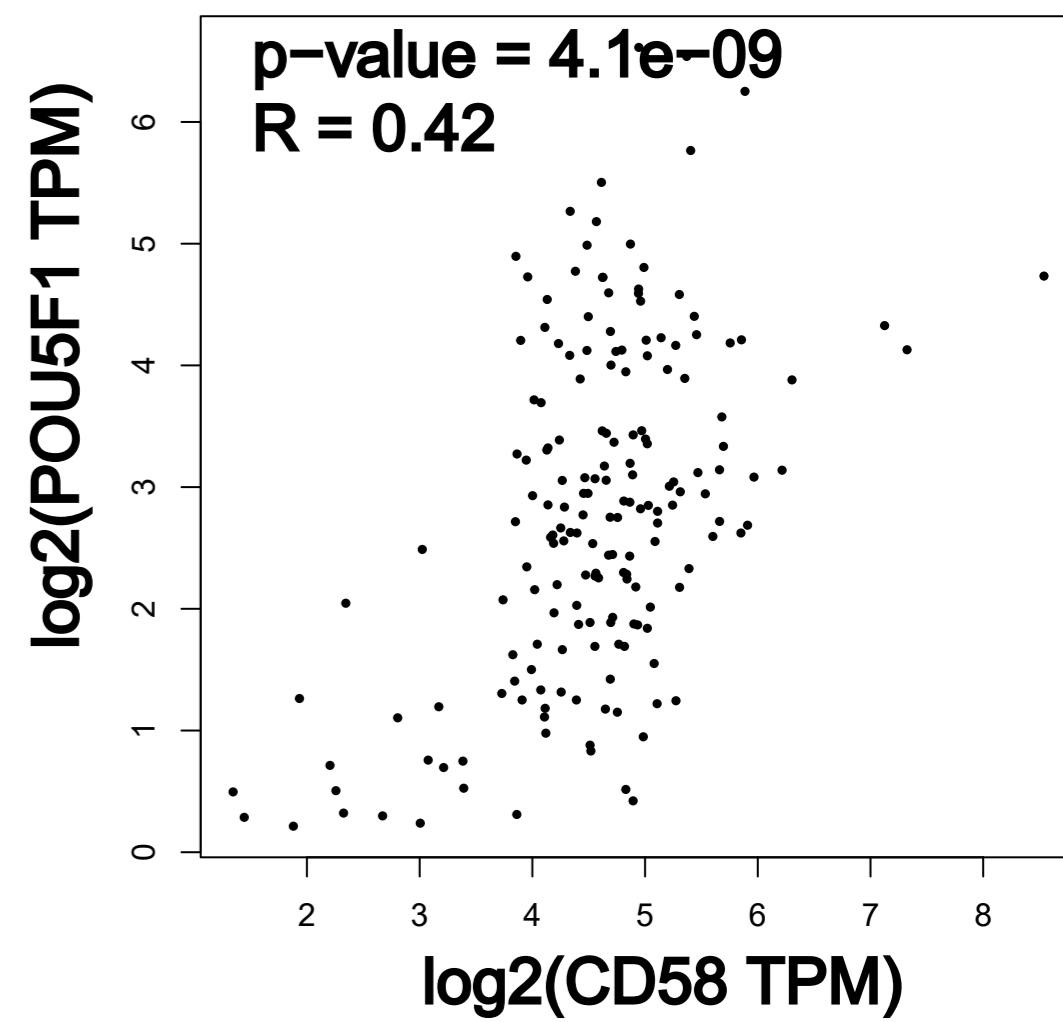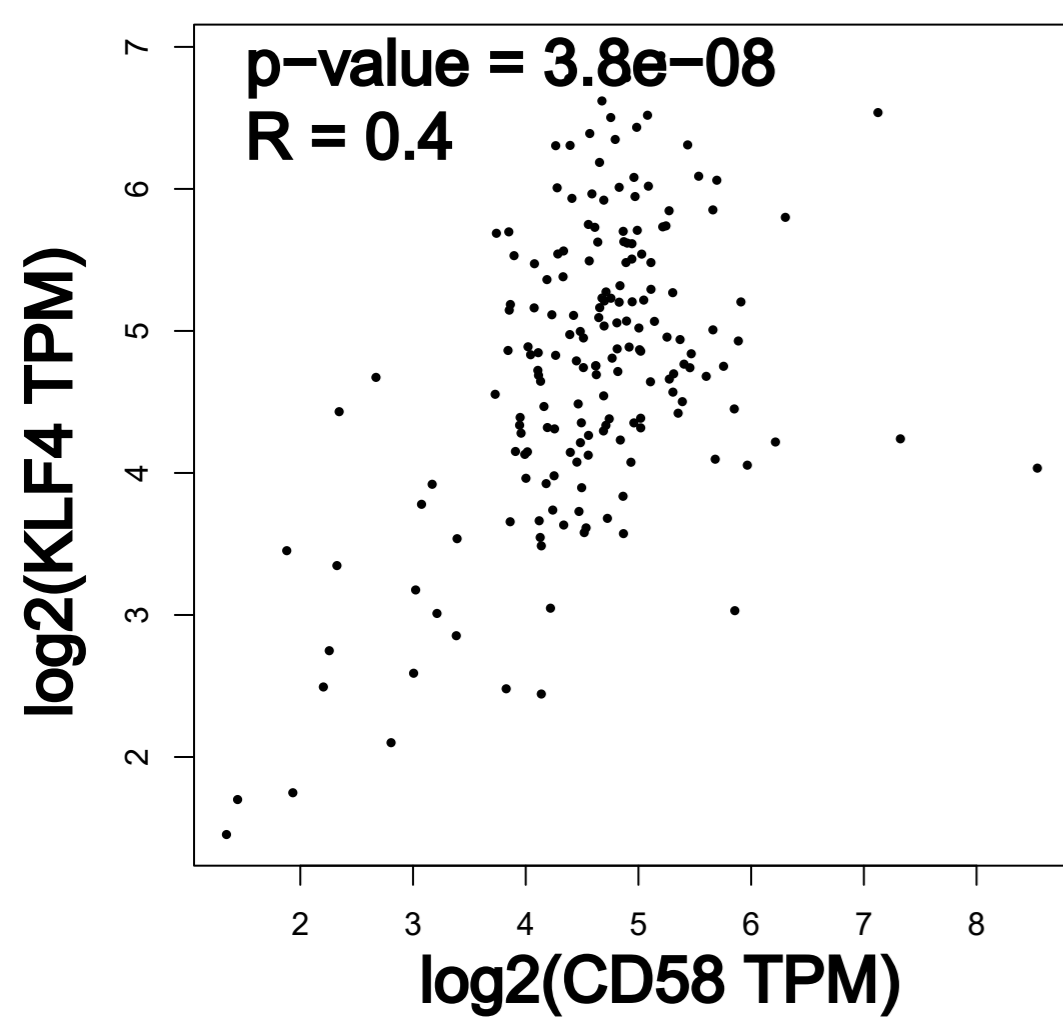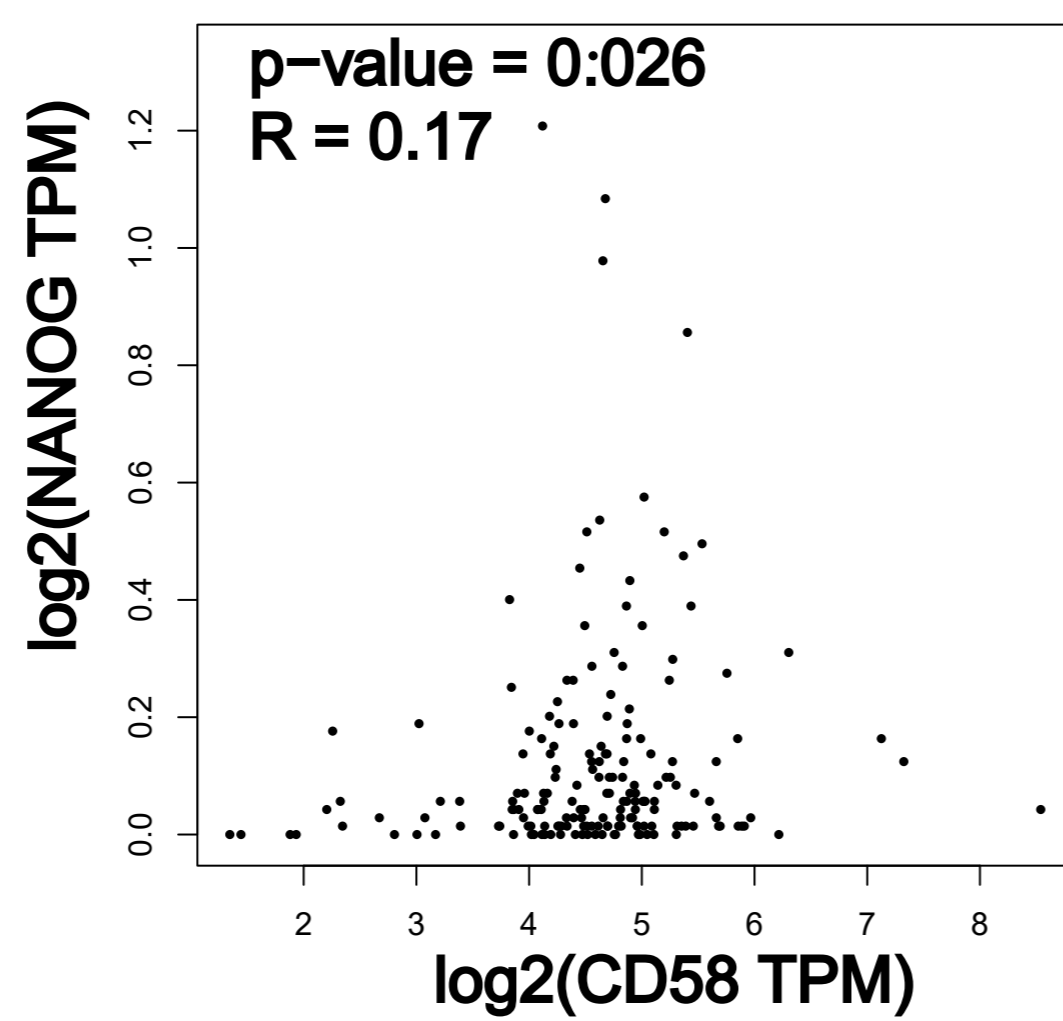

Supplement: Supplementary file 2 — Additional file 2: Figure S2. The relationship between CD58 and immune-related, EMT-related, and CSC-related genes in GEPIA. [file 12935_2021_2037_MOESM2_ESM.pdf]
